# A Non‐Invasive and DNA‐free Approach to Upregulate Mammalian Voltage‐Gated Calcium Channels and Neuronal Calcium Signaling via Terahertz Stimulation

**DOI:** 10.1002/advs.202405436

**Published:** 2024-10-22

**Authors:** Yuankun Sun, Jinli Geng, Yu Fan, Yangmei Li, Yuan Zhong, Jing Cai, Xiaodong Liu, Shaomeng Wang, Yubin Gong, Chao Chang, Yaxiong Yang, Chunhai Fan

**Affiliations:** ^1^ School of Electronic Science and Engineering University of Electronic Science and Technology of China Chengdu 611731 P. R. China; ^2^ Key Laboratory of Biomechanics and Mechanobiology (Beihang University) Ministry of Education Beijing Advanced Innovation Center for Biomedical Engineering School of Biological Science and Medical Engineering Beihang University Beijing Beijing 100191 P. R. China; ^3^ Innovation Laboratory of Terahertz Biophysics National Innovation Institute of Defense Technology Beijing 100072 P. R. China; ^4^ School of Physics Peking University Beijing 100871 P. R. China; ^5^ School of Chemistry and Chemical Engineering Institute of Molecular Medicine Renji Hospital School of Medicine Shanghai Jiao Tong University Shanghai 200240 P. R. China

**Keywords:** calcium signaling, infrared, neuromodulation, terahertz, voltage‐gated calcium channel

## Abstract

Mammalian voltage‐gated calcium channels (Ca_V_) play critical roles in cardiac excitability, synaptic transmission, and gene transcription. Dysfunctions in Ca_V_ are implicated in a variety of cardiac and neurodevelopmental disorders. Current pharmacological approaches to enhance Ca_V_ activity are limited by off‐target effects, drug metabolism issues, cytotoxicity, and imprecise modulation. Additionally, genetically‐encoded channel activators and optogenetic tools are restricted by gene delivery challenges and biosafety concerns. Here a novel terahertz (THz) wave‐based method to upregulate Ca_V_1.2, a key subtype of Ca_V_, and boost Ca_V_1‐mediated Ca^2+^ signaling in neurons without introducing exogenous DNA is presented. Using molecular dynamics simulations, it is shown that 42.5 THz (7.05 µm, 1418 cm^−1^) waves enhance Ca^2+^ conductance in Ca_V_1.2 by resonating with the stretching mode of the ‐COO^−^ group in the selectivity filter. Electrophysiological recordings and Ca^2+^ imaging confirm that these waves rapidly, reversibly, and non‐thermally increase calcium influx of Ca_V_1.2 in HEK293 cells and induce acute Ca^2+^ signals in neurons. Furthermore, this irradiation upregulates critical Ca_V_1 signals, including CREB phosphorylation and c‐Fos expression, in vitro and in vivo, without raising significant biosafety risks. This DNA‐free, non‐invasive approach offers a promising approach for modulating Ca_V_ gating and Ca^2+^ signaling and treating diseases characterized by deficits in Ca_V_ functions.

## Graphical abstract

A terahertz (THz) wave‐based technique is introduced to upregulate mammalian Ca_V_ channels and neuronal Ca^2+^ signaling. The technique, validated by diverse methods including the molecular dynamics simulations, electrophysiological studies, and Ca^2+^ imaging, demonstrates increased calcium influx and activation of key neuronal signaling pathways, offering a novel, non‐invasive, and DNA‐free therapeutic approach for diseases linked to Ca_V_ dysfunction

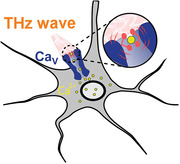

## 1 Introduction

Mammalian voltage‐gated calcium channels (Ca_V_) convert electrical signals from membrane depolarization into calcium signals, mediating synaptic transmission, hormone release, vascular tone, muscle contraction, gene expression, and so on.^[^ ^1^ ^]^ As a representative of the ten mammalian Ca_V_ subtypes (Ca_V_1.1‐1.4, known as the L‐type calcium channels, Ca_V_2.1‐2.3, Ca_V_3.1‐3.3), Ca_V_1.2 is widely expressed in the heart and brain, which initiates the contraction of cardiac and smooth muscle cells, or mediates a variety of Ca^2+^ signaling events in neurons.^[^ ^2^ ^]^ Beyond this, Ca_V_1.2 participates in insulin secretion in pancreatic beta cells,^[^ ^3^ ^]^ and multiple functions in non‐excitable tissues.^[^ ^4^ ^]^ Alterations of the Ca_V_1.2 functions may lead to diverse disorders. For instance, gain‐of‐function variants of Ca_V_1.2 are linked to Timothy Syndrome, which characterized by arrhythmia (prolonged QT interval) and autism.^[^ ^5^ ^]^ In contrast, loss‐of‐function variants of Ca_V_1.2 can also lead to neurodevelopmental abnormalities, epilepsy, autism, short QT syndrome, and so on.^[^ ^6^ ^]^ Small molecule drugs targeting the Ca_V_1 channels have been explored for over half a century and are widely used clinically to treat heart and brain disorders.^[^ ^7^ ^]^ Blockers like isradipine, verapamil, and diltiazem, have been used to treat hypertension, atrial arrhythmias, and migraine headaches, whereas agonists like Bay K 8644 and Baclofen, hold potential for the treatment of ischemic neuronal injury and hearing loss.^[^ ^2^, ^7^, ^8^ ^]^ However, due to the multi‐target nature of the small molecule drugs and the widespread tissue distribution of Ca_V_ channels, applications of these drugs, especially the agonists, carry multiple risks including off‐target effects, drug metabolism, drug dependence, and cytotoxicity.^[^ ^7^, ^9^ ^]^ Treatment of the Ca_V_1 agonists has been reported to induce cell death and cause dysfunctions in the heart and brain of rodents.^[^ ^10^ ^]^ Moreover, small molecule drugs often lack spatiotemporal resolution, limiting their applications in cutting‐edge biological research that demand precise control at the cellular or even subcellular level. While genetically‐encoded channel modulators combined with optogenetic tools have been developed to achieve higher spatiotemporal inhibition of Ca_V_ channels, similar strategies to enhance the Ca_V_ gating and signaling are rarely reported, and their clinical applications are hindered by gene delivery and bio‐safety concerns.^[^ ^11^ ^]^ Hence, an exogenous DNA‐free, reversible, non‐invasive method offering high spatiotemporal upregulation would revolutionize both Ca_V_ channel research and clinical applications.

Structurally, the Ca_V_ channel comprises the pore‐forming α_1_ subunit and auxiliary subunits. The α_1_ subunit itself is composed of 24 transmembrane segments grouped into four repeat domains (DI‐DIV), with each domain containing 6 transmembrane helices (S1‐S6). The S1‐S4 form the voltage sensor domain (VSD), and the S5‐S6 form the pore region. The selectivity filter (SF) within the pore region is pivotal for the ion selectivity and ion conductance.^[^ ^12^ ^]^ It is widely accepted that the carboxyl groups (‐COO^−^) within the key negatively‐charged residues of the SF interact with water molecules surrounding positively‐charged Ca^2+^ ions, forming H‐bonds to achieve high Ca^2+^ ion selectivity. High Ca^2+^ permeation is attributed to the knock‐off effect of electrostatic repulsion when one entering Ca^2+^ ion repels the preceding Ca^2+^ ions within the SF region, emphasizing the importance of the charged functional groups in the SF.^[^ ^13^ ^]^ Since the chemical bonds within the functional groups possess intrinsic vibrational spectra, introducing an external electromagnetic field may alter the bond vibration, thus influencing the ion conductance of the ion channels. Indeed, several research teams, including ours, have theoretically proposed that the ion channels, such as the Ca_V_Ab (an engineered prokaryotic calcium channel), Na_V_Ab (a prokaryotic sodium channel), KcsA (a bacterial potassium channel), and K_V_ (voltage‐gated potassium channel), might respond to the external electromagnetic field exposure by altering their ion conductance.^[^ ^14^ ^]^ Notably, our theoretical study pointed out that the terahertz (THz, 10^12^ Hz) wave boosts Ca^2+^ permeation of Ca_V_Ab through interacting with the SF,^[^ ^14d^ ^]^ suggesting that mammalian Ca_V_ channel, which shares similar Ca^2+^ conductance characteristics with Ca_V_Ab, may also be influenced by the terahertz wave.

In addition to the theoretical modulation of ion channels, the terahertz waves have been experimentally applied to various biological subjects including skin, sperm, neuron, cancer cell, and mouse brain to investigate the biological effects, primarily because of their non‐invasive and exogenous DNA‐free characteristics.^[^ ^14^, ^15^ ^]^ The frequency of applied terahertz waves spans from 0.1 to 100 THz. However, a significant portion of these experimental studies did not analyze the specific regulation of biomacromolecules by the terahertz waves.^[^ ^15^, ^16^ ^]^ It is acknowledged that the rotational and vibrational energy levels of biomacromolecules fall within the terahertz range, implying that the influence of terahertz waves on biomacromolecules, including the ion channels, can be attributed to the resonance.^[^ ^14d,e^ ^]^ Comprehensive studies involving the precise, real‐time electrophysiological recordings of ion channels in combination with the study of molecular dynamics simulations could greatly improve our understanding of the terahertz effects on biomacromolecules, especially the ion channels.

**Figure 1:**
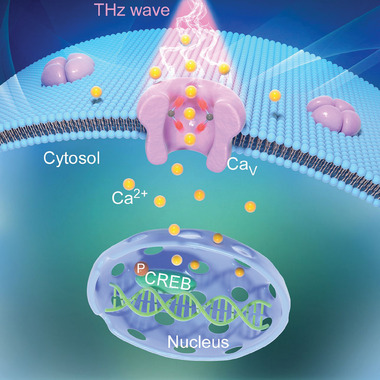
Schematic of THz enhancement of Ca_V_ and neuronal Ca^2+^ signaling. A diagram that showcases the effect of THz wave on mammalian Ca_V_ and neuronal Ca^2+^ signaling. The THz wave resonates with the ‐COO^−^ group in the selectivity filter, thereby promoting the Ca_V_ gating and amplifying the neuronal Ca^2+^ signaling, as manifested by the CREB (cAMP responsive element binding protein, a transcription factor) phosphorylation.

In this work, drawing inspiration from our previous study that theoretically demonstrated the terahertz effects on the Ca_V_Ab,^[^ ^14d^ ^]^ we hypothesized that the THz wave might facilitate the mammalian Ca_V_ channels and enhance the neuronal Ca^2+^ signaling (**Figure** **1**). Our molecular dynamics simulations, focused on Ca^2+^ ion permeation through the Ca_V_ SF with and without the terahertz field, showed that a frequency of 42.5 THz (7.05 µm, 1418 cm^−1^), resonating with the ‐COO^−^, facilitates the Ca^2+^ ion conductance. Whole‐cell patch clamp recordings of recombinant mammalian Ca_V_ channels in HEK293 cells pre, during, and post‐THz confirmed the rapid, reversible, and non‐thermal impacts of the 42.5 THz on Ca_V_. The isolated Ca_V_ currents and related Ca^2+^ signaling pathways in neurons also affirmed this THz modulation pattern. Furthermore, we show in vivo effects of the 42.5 THz on the mouse brain. Collectively, these findings support the idea that the 42.5 THz wave is an exogenous DNA‐free, acute, reversible, and non‐invasive strategy to modulate the Ca_V_ channels with the potential to influence associated pathophysiological events.

## 2 Results

### 2.1 Molecular Dynamics Simulations Reveal the Upregulation of Ca_V_ by a 42.5 THz Wave

**Figure 2:**
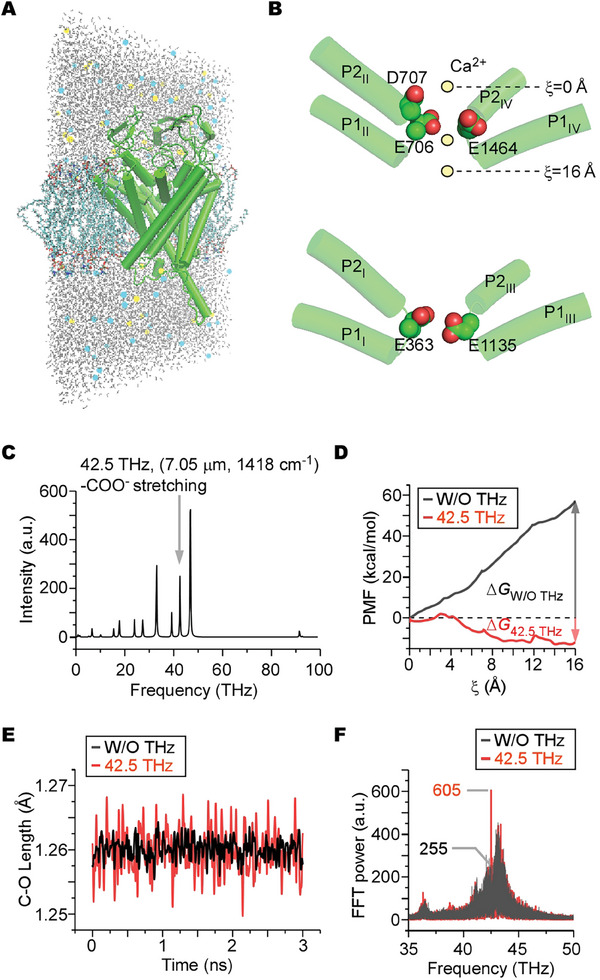
Molecular dynamics simulations of enhanced Ca^2+^ permeation through the Ca_V_1.2 under the 42.5 THz (7.05 µm, 1418 cm^−1^) wave radiation. A) The model building of Ca_V_1.2 pore region (shown as cylinders and colored as green) in a POPC membrane (colored as cyan) via GROMACS. Yellow and blue dots represent the Ca^2+^ ions and Cl^−^ ions, respectively. The H_2_O molecules are colored as gray. B) Side views of the selectivity filter (SF) of Ca_V_1.2, formed by the P1 and P2 helices. The upper panel shows helices from DII and DIV, while the bottom panel shows helices from DI and DIII. Key negatively‐charged amino acids are labeled and shown as spheres. Ca^2+^ ions (colored as yellow) are shown to illustrate the positions of ξ. C) The vibrational/absorption spectrum of the carboxyl and carbonyl groups. The peak absorption of symmetric ‐COO^−^ stretching is labeled. D) Free energy profiles without (black curve) or with (red curve) the presence of external 42.5 THz fields. E) The C─O bond length oscillations in the ‐COO^−^ group without (black curve) or with (red curve) the presence of the 42.5 THz field. F) Fast Fourier Transform (FFT) was applied to the two oscillation curves of varying lengths. The FFT power for the red curve (exposed to 42.5 THz field) at 42.5 THz is 605 a.u., in contrast to the black curve (not exposed to the THz field) which registered 255 a.u. at 42.5 THz.

The Ca_V_1.2 channel consists of the pore‐forming α_1C_ subunit and auxiliary subunits. Using both the AlphaFold predicted structure and Cryo‐EM‐solved structure,^[^ ^17^ ^]^ we constructed a simplified mode of the pore region of a human α_1C_ formed by the S5‐pore helix‐S6 using the GROMACS (**Figure** **2**A; Figure S1, Supporting Information). By simulating a Ca^2+^ ion's transit through the pore, we estimated the potential of mean force (PMF) using umbrella sampling (US) methods (Figure S2, Text S1, and Table S1, Supporting Information). The free energy profile demonstrates a significant energy barrier for Ca^2+^ to traverse the SF, with a ΔG of 59 kcal mol^−1^. Within the SF, two key calcium ion‐selective sites, termed Site1 and Site2, are noted to limit the Ca^2+^ movement. Site1 contains residue D707, while Site2 is formed by residues E363, E706, E1135, and E1464 (Figure 2B). The functional groups of the amino acids manifest bond absorptions at different locations and intensities in the THz spectrum. After Fourier transforming the electrical flux‐flux autocorrelation function, we calculated the absorption spectra of the carboxyl and carbonyl groups within the SF and detected a pronounced peak at 42.5 THz (7.05 µm, 1418 cm^−1^), which is corresponding to the symmetric stretching mode of the ‐COO^−^ group within the negative‐charged amino acids (Figure 2C).^[^ ^14d^ ^]^ When the SF was subjected to an external 42.5 THz field, the PMF profile underwent a significant transformation, revealing a ΔG of ‐11 kcal mol^−1^ (Figure 2D). This dramatic shift, stemming from increased oscillations of the C─O bond length within the ‐COO^−^ group (Figure 2E) and resonance‐mediated amplification of vibrations at 42.5 THz (Figure 2F), indicates the potential of the 42.5 THz wave to facilitate Ca_V_1.2 channel gating and boost Ca^2+^ ion influx.

Mammalian Ca_V_1.2 and Ca_V_Ab manifest substantial distinctions in both amino acid sequences and the pore‐forming patterns (Figure S3A, Supporting Information). The SF of Ca_V_1.2 is constituted by the four homologous repeat domains (I–IV) of α_1C_, while the SF of Ca_V_Ab is composed of four homotetrameric subunits. The overall structures of the two channels are markedly distinct, with a root‐mean‐square deviation (RMSD) of 10 Å. However, despite these differences, the SF regions of both channels display similarity, characterized by negatively charged amino acids in the core regions of SF and comparable pore radii (Figure S3B, Supporting Information). The resemblance in the SF regions underscores the consistent enhancement of the Ca^2+^ conductance induced by the 42.5 THz wave. Furthermore, a comprehensive comparison was conducted across the ten mammalian Ca_V_ subtypes. Multiple sequence alignment of the SF regions within the Ca_V_ family reveals a high degree of conservation (Figure S3A, Supporting Information). Additionally, the overall architecture of the α_1C_ is akin to all other representative Ca_V_ subtypes (Ca_V_1.1, Ca_V_1.3, Ca_V_1.4, Ca_V_2.2 representing Ca_V_2, and Ca_V_3.1 representing Ca_V_3), with RMSD values not exceeding 2 Å. Structural comparisons of the SF regions between Ca_V_1.2 and other Ca_V_ channels indicate higher similarities when juxtaposed with the disparities between Ca_V_1.2 and Ca_V_Ab (Figure S3B–G, Supporting Information). Collectively, these pieces of evidence suggest a conserved mechanism of THz‐mediated regulation of Ca^2+^ permeation across the Ca_V_ family.

Ca_V_ channels, alongside sodium (Na_V_), potassium (K_V_), and TRP channels, are expressed in excitable cells. A critical question is whether 42.5 THz irradiation could influence these various channels. Our initial approach involved a thorough comparison of Ca_V_ channels with representative Na_V_, K_V_, and TRP channels (Figure S3A, Supporting Information). A detailed sequence alignment of the selectivity filter (SF) regions across these channels highlighted that K_V_ and TRP channels lack negatively‐charged amino acids in the SF. Moreover, the structural differences between these channels and Ca_V_1.2 suggest a lack of responsiveness to 42.5 THz stimuli. Na_V_ channels, containing two negatively‐charged amino acids in the SF and bearing structural similarities to Ca_V_ channels (Figure S3A, Supporting Information), were also evaluated. However, previous research indicated that Na_V_ channels' vibrational spectra do not exhibit resonance peaks at 42.5 THz,^[^ ^14e^ ^]^ implying that 42.5 THz waves likely do not influence Na_V_ channel activity. Thus, it appears that 42.5 THz irradiation does not significantly impact the function of Na_V_, K_V_, and TRP channels.

### 2.2 Electrophysiological Validation of the 42.5 THz Effect on Recombinant Ca_V_

**Figure 3:**
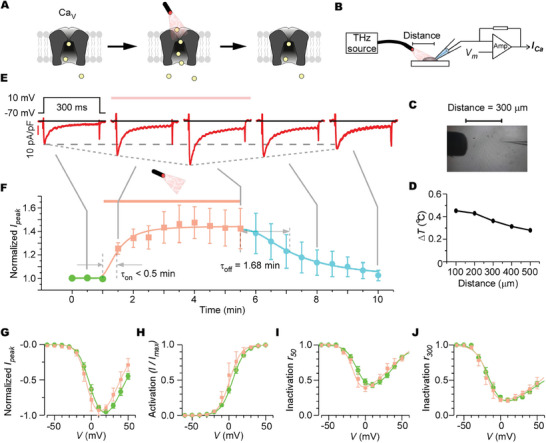
Electrophysiological recording of recombinant Ca_V_1.2 under the 42.5 THz wave exposure. A) Schematic of patch‐clamp recording of Ca_V_1.2 in HEK293 cells to compare the gating properties pre, during, and post‐THz stimulation. B) Configuration for patch‐clamp recording. The distance between the optical fiber port and the glass electrode is ≈300 µm. C) Representative image to show the configuration of patch‐clamp recording. The distance between the optical fiber port and the glass electrode is 300 µm. D) The relationship between the distance and the temperature elevation under 10 min irradiation of 42.5 THz wave in the experimental setting. E) Representative traces of recombinant Ca_V_1.2 pre, during, and post THz stimulation. 300 ms Ca^2+^ currents were evoked at 30 s intervals by steps from −70 to +10 mV. F) Temporal profile of the peak Ca^2+^ currents (n = 9 cells). Data points corresponding to pre, during, and post‐THz stimulation are colored by green, pink, and cyan, respectively. G, H) The current–voltage (*I–V*) curves (G) and the voltage dependence of activation curves (H) of Ca_V_1.2 Ca^2+^ currents without (green dots, n = 11 cells) or with (pink square markers, n = 4 cells) the THz stimulation. I,J) The inactivation profiles of *r*_50_ (I) and *r*_300_ (J) of Ca_V_1.2 Ca^2+^ currents without (green dots, n = 11 cells) or with (pink square markers, n = 4 cells) the THz stimulation. *r*_50_ = *I*_50 ms_/*I*_peak_, and *r*_300_ = *I*_300 ms_/*I*_peak_. Values are presented as mean±SEM.

To assess the actual impact of 42.5 THz waves on Ca_V_, we reconstituted the human Ca_V_1.2 channels in HEK293 cells for electrophysiological recordings (**Figure** **3**A). Compared to native cells expressing endogenous Ca_V_1.2 channels, HEK293 cells expressing recombinant Ca_V_1.2 minimize the potential disturbances from other membrane ion channels and receptors,^[^ ^18^ ^]^ making them an ideal platform for testing the THz effect on Ca_V_. The 42.5 THz stimulation emanated from a customized laser source, generating a power density of roughly 0.066 µW µm^−2^ at the laser fiber optic port (Figure 3B; Figure S4A, Supporting Information). During recordings, the fiber optic port was positioned 300 µm from the patch clamp pipette to minimize potential thermal effects (Figure 3C), even though minor water absorption was observed at this frequency.^[^ ^14e^ ^]^ To rigorously exclude the thermal influence, we measured temperature fluctuations during a 10‐min 42.5 THz irradiation from different distances, utilizing a temperature probe to record local temperature changes (Figure S4B–D, Supporting Information). The derived relationship between distance and temperature rise confirmed a negligible temperature elevation (<0.5 °C) (Figure 3D). Concurrently, throughout the patch‐clamp recordings, we perfused bath solutions, ensuring a uniform temperature milieu of the cells. Within this framework, we performed the whole‐cell voltage‐clamp to record the Ca^2+^ currents of recombinant Ca_V_1.2 pre‐, during, and post‐THz exposure (Figure 3E). The Ca^2+^ currents through Ca_V_1.2, evoked at 30 s intervals by steps to +10 mV from −70 mV holding potential, were rapidly enhanced over 40% under THz stimulation, showcasing a τ_on_ (time to attain half‐maximal response) of less than 30s. The THz‐mediated enhancement persisted throughout several minutes of stimulation and receded to baseline levels with a τ_off_ (time to revert to half‐maximal response) of 1.68 min post‐stimulation (Figure 3F). By evaluating the normalized current‐voltage (*I*‐*V*) profiles and the voltage dependence of activation profiles both pre and during THz exposure (Figure 3G,H), we discerned only a non‐significant shift to the left in the voltage‐dependent activation under THz (≈5 mV discrepancy at half‐maximal activation), refuting potential THz‐driven depolarization, and corroborating the conclusions from molecular dynamics simulations. Acknowledging that Ca_V_1.2 and other Ca_V_ channels manifest pronounced calcium‐dependent inactivation (CDI), we also assessed CDI at 50 ms and 300 ms of the step stimulation. We detected no significant CDI alterations when compared to the Ca^2+^ currents before or during THz exposure (Figure 3I,J), reinforcing the conclusion of augmented Ca^2+^ influx under THz stimulation.

### 2.3 Validation of the 42.5 THz Effect on Ca_V_1‐Mediated Neuronal Ca^2+^ Signaling

**Figure 4:**
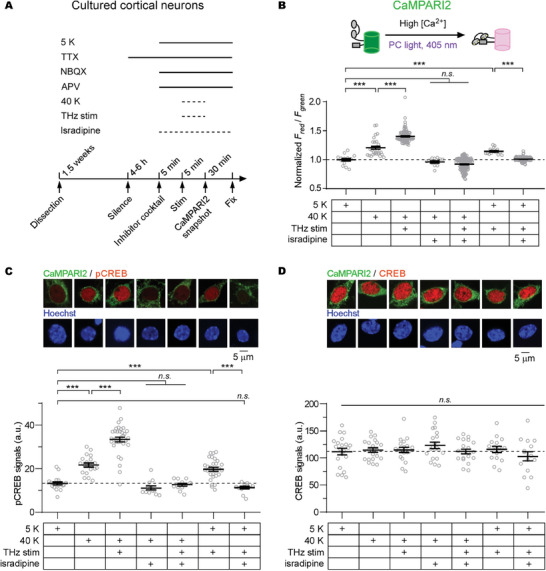
The 42.5 THz wave impact endogenous Ca_V_1 and neuronal Ca^2+^ signaling. A) Detail of the experimental protocol for THz stimulation on endogenous Ca_V_1 channels in cultured cortical neurons. TTX (Tetrodotoxin), sodium channel blocker, final concentration: 0.5 µm. NBQX, AMPA receptor blocker, 10 µm. APV, NMDA receptor blocker, 10 µm. Isradipine, Ca_V_1 channel blocker, 10 µm. B) A schematic to illustrate the working mechanism of the genetically‐encoded calcium integrator CaMPARI2. Coincidental Ca^2+^ and PC light drive the irreversible green‐to‐red conversion of CaMPARI2. Statistical summary of normalized *F*_red_/*F*_green_ is shown to quantify the total calcium influx of neurons under different stimuli labeled below (n = 15, 31, 101, 75, 100, 58, and 100 cells for 7 columns from left to right). C) Statistical summary of the pCREB signals (n = 20, 20, 35, 12, 12, 28, and 12 cells for 7 columns from left to right). Representative images of merged channels with CaMPARI2 fluorescence (green) and pCREB immunofluorescence (red), and hoechst (blue) for nuclear staining are shown. D) Statistical summary of CREB signals (n = 20, 20, 20, 20, 20, 15 and 15 cells for 7 columns from left to right). Representative images of merged channels with CaMPARI2 (green) and CREB (red), and Hoechst channel (blue) are shown. Values are presented as mean±SEM. One‐way ANOVA followed by Tukey for post hoc tests were used (^***^ *p* < 0.001; *n.s*., not significant, *p* > 0.05).

Ca_V_1.2 channels are prominently expressed in neurons, playing a pivotal role in neuronal Ca^2+^ signaling.^[^ ^1a^ ^]^ We isolated neurons from the mouse cortex to assess the impact of THz on Ca_V_1‐mediated neuronal Ca^2+^ signaling. TTX, NBQX, and APV were applied to silence the neurons and block the Ca^2+^‐permeable AMPA receptors and NMDA receptors, respectively. The induction of Ca^2+^ flux, predominantly through Ca_V_1, was efficiently triggered by a 40 K stimulus.^[^ ^19^ ^]^ Furthermore, the specific Ca_V_1 inhibitor, isradipine, was employed to validate these Ca_V_1 currents (**Figure** **4**A). An optimized genetically‐encoded calcium integrator, CaMPARI2, was utilized to quantify the cumulative calcium influx following stimulation, allowing a temporally precise “snapshot” of neuronal Ca^2+^ signals.^[^ ^20^ ^]^ CaMPARI2 undergoes irreversible green‐to‐red conversion in the presence of Ca^2+^ and concurrent PC light (405 nm) illumination. Consequently, the *F*_red_ to *F*_green_ ratio becomes a metric indicating the total calcium influx. Cultured cortical neurons virally‐expressing CaMPARI2 exhibited a significant increase in the normalized *F*_red_/*F*_green_ ratio when subjected to a 40 K stimulus. This effect was further intensified under concurrent THz stimulation. Additionally, the THz stimulation on its own triggered calcium influx in a 5 K environment, likely due to the amplification of the spontaneous regenerative calcium transients of Ca_V_1.^[^ ^21^ ^]^ Treatment of isradipine suppressed all the observed calcium signals, confirming that THz effectively amplifies Ca^2+^ signals through endogenous Ca_V_1 channels in neurons (Figure 4B). CREB (cAMP responsive element binding protein, a transcription factor) signaling pathway represents one of the key downstream Ca^2+^ signals in neurons.^[^ ^22^ ^]^ Upon silencing the neurons and inhibiting AMPA receptors and NMDA receptors, the CREB signaling cascade is predominantly initiated by Ca_V_ channels.^[^ ^19^ ^]^ Maintaining consistency with the CaMPARI2 experiment, we evaluated Ca_V_1‐mediated CREB phosphorylation and noted congruent outcomes. THz stimulation elevated pCREB levels, an effect that was neutralized by isradipine (Figure 4C). Isradipine alone did not alter pCREB levels compared to the unstimulated group, indicating that the inhibition of THz‐driven pCREB upregulation occurs through the modulation of neuronal Ca_V_1 signals (Figure S5A, Supporting Information). Meanwhile, total CREB quantities remained consistent across all experimental conditions, implying that THz stimulation has an immediate impact on CREB phosphorylation without influencing long‐term CREB expression (Figure 4D; Figure S5B, Supporting Information). These findings collectively reinforce the conclusion that THz stimulation robustly amplifies the neuronal Ca^2+^ signaling downstream of the activities of endogenous Ca_V_1 channels.

### 2.4 The Effects of the 42.5 THz Wave in Cultured Neurons

**Figure 5:**
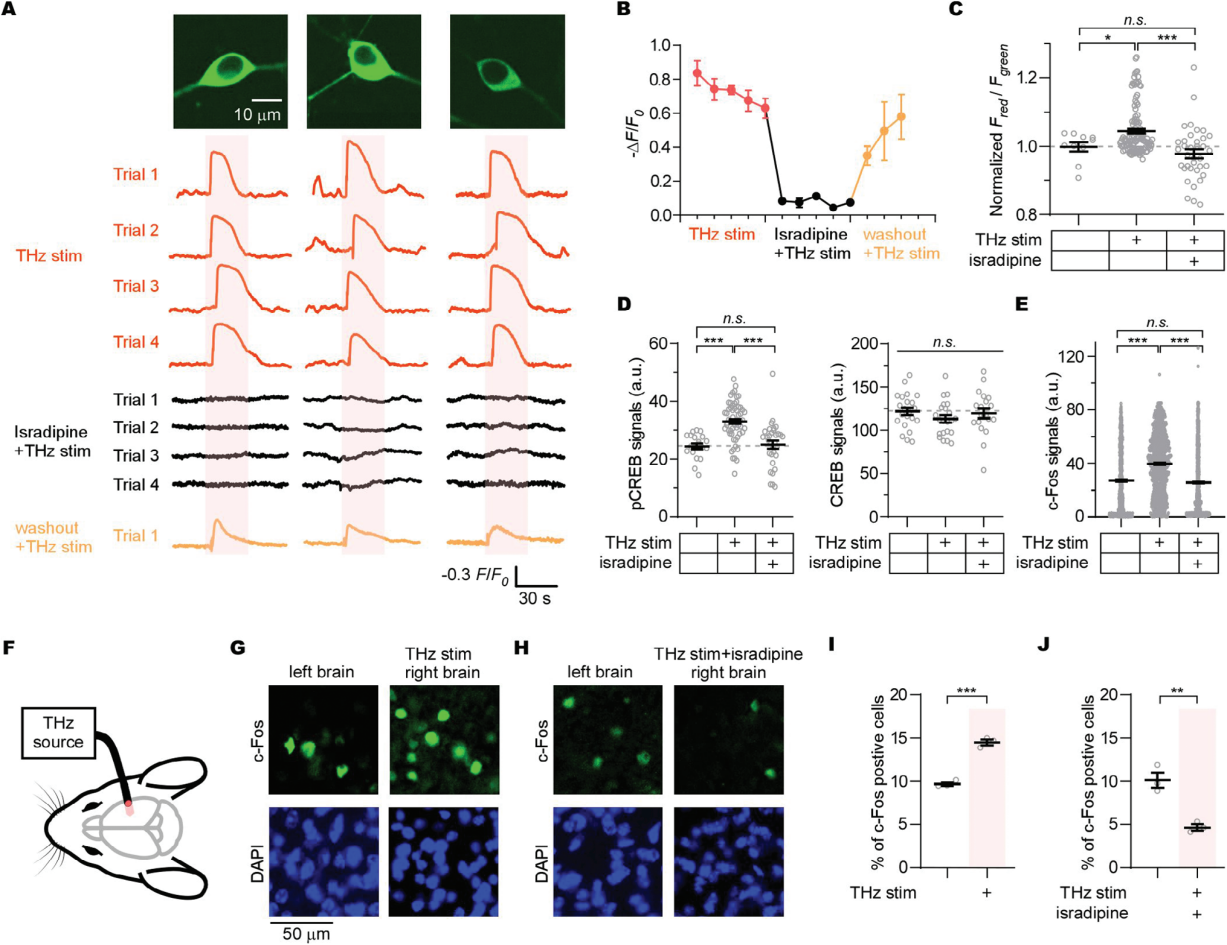
Applications of 42.5 THz‐based channel/neuro‐modulation in vitro and in vivo. A) The traces of calcium images for 3 representative cultured cortical neurons virally‐expressing CaMPARI2 under 3 conditions: THz stimuli under physiological condition (traces in red), THz stimuli under isradipine treatment (traces in black), and THz stimuli post washout of isradipine (traces in yellow). The orange bars mark the duration of the THz stimuli. B) Statistical summary of the peak amplitude of neurons virally‐expressing CaMPARI2 under the stimuli in (A). C) Statistical summary of total calcium influx of neurons virally‐expressing CaMPARI2 under 3 conditions: basal/physiological condition (n = 10 cells), THz stimuli (n = 131 cells), and THz stimuli under isradipine treatment (n = 36 cells). The normalized *F*_red_/*F*_green_ of CaMPARI2 serves as the index. D) Statistical summary of pCREB signals (n = 20, 59, and 32 cells) and CREB signals (n = 22, 22, and 20 cells) of neurons under the identical 3 conditions. All signals are immunofluorescent signals in neurons. E) Statistical summary of c‐Fos immunofluorescent signals of neurons (n = 809, 959, and 1039 cells) under the identical 3 conditions. F) Diagram detailing THz stimulation applied to the right motor cortex in anesthetized mice, with the left cortex as a control. G) Representative images of c‐Fos immunofluorescences in brain slices from the same mice under two conditions: left brain without the THz stimulation (left images), and right brain with the THz stimulation (right images). DAPI is used for nuclear staining and cell counting. H) c‐Fos immunofluorescence images of brain slices under two conditions: left brain without THz stimulation (left images), and right brain of the same mice with isradipine pre‐treatment before THz exposure (right images). DAPI is used for nuclear staining and cell counting. I,J) Statistical summaries of c‐Fos immunofluorescences in brain slices from the same three mice, corresponding to conditions described in (G) and (H), respectively. Values are presented as mean±SEM. Student's unpaired *t*‐test (I, J), one‐way ANOVA followed by Tukey for post hoc tests (B–D), and Kruskal–Wallis test (nonparametric test) (E) were used (^*^ *p* < 0.05, ^**^ *p* < 0.01, ^***^ *p* < 0.001; *n.s*., not significant, *p* > 0.05).

Next, we evaluated the impact of 42.5 THz on cultured cortical neurons under physiological conditions to explore the potential applications. **Figure** **5**A showcases three representative neurons virally‐expressing CaMPARI2. These neurons were cultured for 1.5 weeks and were maintained in a 5 K solution before Ca^2+^ imaging without any drug treatment. The isolated neurons were observed to be rapidly, reversibly, and repeatedly activated by 42.5 THz stimulation, resulting in significant calcium influx. While the neurons exhibited spontaneous Ca^2+^ activities at the basal condition, the amplitudes and durations were significantly lower than the THz‐evoked Ca^2+^ activities. Next, we acutely applied 10 µm isradipine to neurons for 5 min, and found that the 42.5 THz failed to evoke detectable calcium signals; however, washing out the isradipine partially restored the calcium response (Figure 5B), suggesting that the THz‐mediated enlargement of acute Ca^2+^ signals in native neurons under physiological condition is mainly through the Ca_V_1 channels. Consequentially, downstream events—calcium accumulation evaluated by CaMPARI2, pCREB signals, and the expression of CREB downstream genes, particularly the immediate early gene of c‐Fos as the marker of neuronal activity—increased under THz stimulation. Predictably, all these increases were inhibited by isradipine, while the CREB signals remained unchanged (Figure 5C–E; Figure S6A,B, Supporting Information). While isradipine is known as a specific blocker of Ca_V_1 channels, the possibility of it affecting other targets exists. To address this concern, we evaluated the effects of three other Ca_V_1 channel blockers—nimodipine, diltiazem, and nifedipine—on pCREB signaling under 42.5 THz stimuli. The outcomes were similar to those observed with isradipine (Figure S6C, Supporting Information). These findings collectively highlight the capacity of 42.5 THz irradiation to modulate neuronal activity and enhance the expression of key genes via Ca_V_1‐CREB signaling pathways. Cultured hippocampal neurons demonstrated similar responses, both to the THz stimulation and the isradipine treatment (Figure S7, Supporting Information), suggesting a universal mechanism in all Ca_V_1‐expressing neurons.

### 2.5 Application of the 42.5 THz Wave to Mouse Brain

Since the 42.5 THz wave showed remarkable upregulation of Ca_V_1 channel gating and neuronal Ca^2+^ signaling, we expanded our investigation from cultured neurons to the whole mouse brain to expand the biological implications of THz‐mediated channel/neuro‐modulation. Anesthetized mice were subjected to 42.5 THz stimulation, targeting the right motor cortex, while the left hemisphere served as an internal control (Figure 5F). The THz‐stimulated areas in the right hemispheres exhibited a marked increase in c‐Fos positive cells compared to their unstimulated counterparts in the left hemispheres. And treatment of isradipine before THz stimulation led to a significant decline in these c‐Fos positive cells (Figure 5G–J). These findings substantiate the notion that 42.5 THz can effectively trigger the neural activities and gene expressions presumably mediated by Ca_V_1 channels within the mouse brain.

Venturing beyond the neural activity and gene expression effects, we gauged the immediate behavioral responses induced by THz waves in the awake mouse brain (Figure S8A, Supporting Information). Direct THz stimulation of the motor cortex elicited noticeable trembling in the mouse, as a behavioral response of upregulated neural activities in the motor cortex.^[^ ^23^ ^]^ These mice exhibited a significant, reversible, and repeatable increase in trembling frequency responding to the three consecutive stimulations (Figure S8B, Supporting Information), underscoring the potential of 42.5 THz in neuromodulation and clinical applications.

### 2.6 Biosafety Assessments of 42.5 THz Irradiation

**Figure 6:**
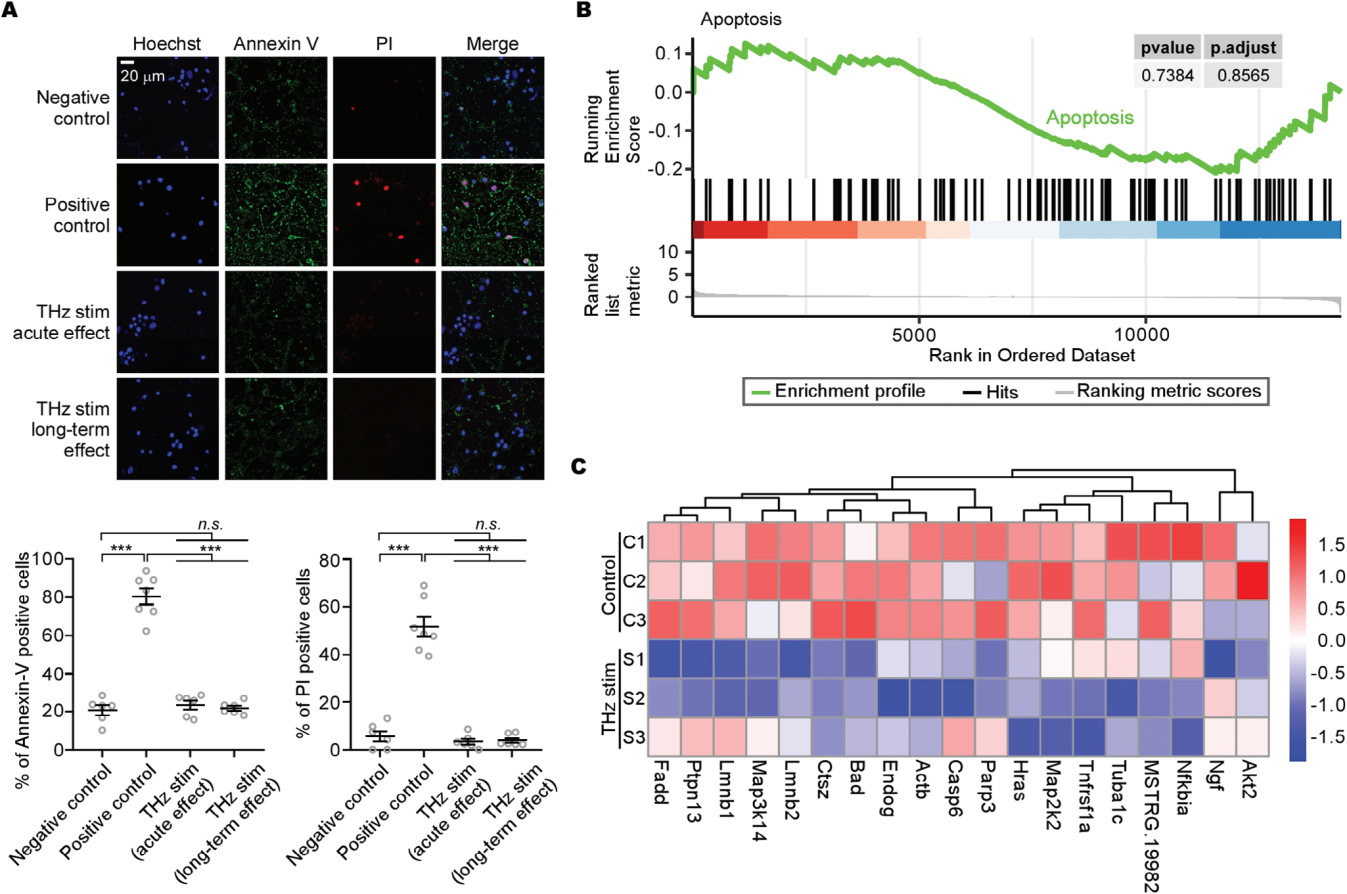
Biosafety Assessments of 42.5 THz‐based channel/neuro‐modulation in vitro and in vivo. A) The Annexin V‐EGFP/PI staining to detect the apoptosis in cultured neurons. Neurons exposed to 42.5 THz for 5 min and analyzed after 2 h or 1 day represent acute and long‐term effects groups, respectively. Nuclei are stained with Hoechst (blue). The percentages of cells positive for Annexin‐V (green, indicating apoptotic cells) and PI (red, indicating dead cells) in each group are statistically summarized. B,C) RNA was extracted from the motor cortex of three irradiated and three control mice one day following a 10‐min exposure to 42.5 THz radiation. Subsequent Gene Set Enrichment Analysis (GSEA) (B) along with heatmap visualization of gene expression (C) demonstrated that the apoptosis pathway remained unaffected. Values are presented as mean ± SEM. One‐way ANOVA followed by Tukey for post hoc tests were used (^***^ *p* < 0.001; *n.s*., not significant, *p* > 0.05).

Before the widespread use of 42.5 THz for modulating channels and neuronal activity, we carefully assessed its biosafety both in vitro and in vivo. We examined the apoptosis in cultured neurons using an Annexin V‐EGFP/PI staining kit (**Figure** **6**A), revealing no significant apoptosis or death no matter for the acute or long‐term effect of 5 min treatment of 42.5 THz stimuli. For a comprehensive understanding of 42.5 THz's impact on mouse brains, RNA from the motor cortex of irradiated and control mice was sequenced a day post 10‐min exposure to 42.5 THz. RNA‐seq data indicated alterations in multiple signaling pathways, including those related to calcium signaling (Figure S9, Supporting Information). The apoptosis pathway, in particular, showed no significant upregulation after GSEA analysis (Figure 6B,C). Overall, these findings suggest that 42.5 THz irradiation at a power level of 0.066 µW µm^−2^ in this study poses no significant biosafety issues, supporting its potential for clinical application.

## 3 Conclusion

In our multifaceted investigation, we delved from molecular dynamics simulations to recombinant system validation, ultimately exploring the effects of neurons both in vitro and in vivo. Our results robustly indicate that the 42.5 THz wave can positively modulate mammalian Ca_V_1.2 channels and enhance neuronal Ca^2+^ signaling. Furthermore, the potentials of 42.5 THz in neuromodulation, regulation of gene expression, and manipulation of animal behavior are profound. The rapid, reversible, repeatable, exogenous DNA‐free, non‐thermal, and biocompatible attributes of 42.5 THz stimulation position it as an innovative technique for channel/neuro‐modulation, and a promising intervention of Ca_V_‐related pathophysiology.

Centered on Ca_V_1.2, our findings reveal that amplifying the vibrations of the ‐COO^−^ groups in negatively charged amino acids within the selectivity filter via the THz wave boosts the calcium ion conduction capability. Given the high conservation of the selectivity filter across Ca_V_ channels,^[^ ^12a^ ^]^ we speculate that other members of the Ca_V_ family might also be influenced by 42.5 THz waves. For instance, Ca_V_1.3, another member of the Ca_V_1 family expressed in neurons, functions nearly identically to Ca_V_1.2.^[^ ^24^ ^]^ Therefore, THz modulation of Ca_V_1.3 would likely result in the same upregulation of neuronal Ca^2+^ signaling. While the Ca_V_2 family could be considered, their contribution, especially to CREB signaling, is seemingly less pivotal compared to Ca_V_1, due to their higher activation voltage and tenuous interactions with calcium signaling proteins.^[^ ^19^, ^25^ ^]^ It is notable that the Ca_V_1 channel blocker isradipine nearly blocked all THz‐evoked Ca^2+^ signals of isolated neurons in physiological conditions, we infer this effect predominantly arises from the spontaneous activities of Ca_V_1 channels, rather than those of Ca_V_2 channels, in the basal state of neurons.^[^ ^21^ ^]^ Given that Ca_V_2 channels play a role in regulating the synaptic transmission and considering that isolated neurons over 1.5 weeks do not form a mature network to emphasize the function of Ca_V_2,^[^ ^25^, ^26^ ^]^ the THz wave likely primarily amplifies the Ca_V_1 signals, leading to the pronounced effect of isradipine. The influence of 42.5 THz on Ca_V_2 channels, especially in the context of synaptic transmission, remains an avenue for future exploration.^[^ ^1a^ ^]^ The Ca_V_3 channels, also known as the T‐type calcium channels, are therapeutic targets of brain disorders.^[^ ^27^ ^]^ While Ca_V_3 might not directly modulate the neuronal CREB signaling,^[^ ^1a^ ^]^ its activation contributes to neuronal firing.^[^ ^28^ ^]^ In this study, we could not rule out the possibility that THz modulation of mice trembling frequency could be linked to an upregulation of Ca_V_3, especially since the Ca_V_3 channels are implicated in tremors and other hyperexcitable phenomena.^[^ ^28^, ^29^ ^]^ The 42.5 THz mediated neuro‐modulation might arise from a combined upregulation of all the Ca_V_ channels present in neurons.

The terahertz frequency band lies within the vibrational spectrum of numerous biomacromolecules.^[^ ^15g^ ^]^ Although our study has highlighted the impact of 42.5 THz on Ca_V_1.2, it is imperative to acknowledge that the vast realm of biomolecules, beyond just Ca_V_, may potentially exhibit nuanced interactions with this frequency. Understanding the unique interaction between the 42.5 THz wave and Ca_V_ channels is essential to address concerns about specificity. First, the 42.5 THz frequency corresponds to the symmetric vibrational frequency of the carboxylate (‐COO^−^) groups of negatively charged amino acids located in the selectivity filters of Ca_V_ channels. Second, these ‐COO^−^ groups play a pivotal role in the passage of calcium ions through the channel, directly affecting the channel's function. Therefore, if other biomacromolecules contain ‐COO^−^ groups that are critical for their normal functioning, the 42.5 THz wave may interact with them. For instance, calmodulin, a calcium‐binding protein, contains multiple negatively charged residues in its EF‐hand motifs that interact with Ca^2^⁺ ions.^[^ ^30^ ^]^ The 42.5 THz wave could potentially resonate with the ‐COO^−^ groups in calmodulin, affecting its calcium‐binding properties. Additionally, charged groups, including carboxylate groups, can participate in hydrogen bonding within protein‐protein interactions,^[^ ^31^ ^]^ which may also be influenced by 42.5 THz radiation. It is important to note that the vibrational frequency of chemical bonds can be shifted depending on the local environment, with shifts ranging from several to a few hundred cm^−1^.^[^ ^32^ ^]^ Therefore, whether the ‐COO^−^ groups in other biomacromolecules resonate exactly at 42.5 THz needs to be analyzed on a case‐by‐case basis. From this perspective, while there is potential for the 42.5 THz wave to affect other biomacromolecules, the specificity may not be overly compromised due to the unique resonance conditions required. Nonetheless, we acknowledge the possibility of off‐target effects.

Moreover, neurons are involved in a wide variety of physiological processes to execute diverse functions. Our RNA‐seq data indicate that 42.5 THz affect multiple signaling pathways in the mouse brain (Figure S9C, Supporting Information), also suggesting a wide range of biomolecules may response to 42.5 THz stimuli. Future studies employing techniques like single‐cell and spatial multi‐omics analyses,^[^ ^33^ ^]^ in combination with electrophysiological and neurochemical approaches, are anticipated to further elucidate the impact of 42.5 THz on these biomacromolecules within neuronal systems. This can also aid in discerning the advantages and limitations of 42.5 THz‐mediated channel/neuro‐modulation.

In addition to concerns about the molecular specificity of 42.5 THz stimulation, localized thermal effects represent another issue that may limit its further application. In our previous study.^[^ ^14e^ ^]^ We measured the temperature at the tip of the optical fiber and found that local temperatures could rise by several degrees Celsius after several minutes of continuous stimulation. To mitigate any localized thermal impacts in our current experiments, we maintained a distance between the optical fiber and the biological tissues, avoiding direct contact, and strictly controlled the irradiation time and intensity. Under our experimental conditions, when the distance between the optical fiber and the biological tissues was greater than 100 µm, the temperature increase was less than 0.5 °C. This minimal temperature rise is unlikely to cause significant thermal effects on the cells or channels. Additionally, the KEGG pathway classification analysis in Figure S9 (Supporting Information) indicated that thermogenesis‐related signaling pathways were not significantly activated. These findings suggest that under our experimental conditions, the cells in the stimulated area did not undergo significant thermal changes. Nonetheless, we acknowledge that if THz stimulation is applied for longer durations, at higher intensities, or in closer proximity to biological tissues, thermal effects could become significant and potentially cause damage. Therefore, further applications of THz stimulation in vivo should carefully control the experimental settings to avoid potential thermal damage.

Apart from the 42.5 THz wave, there's evidence suggesting that other frequencies of THz waves can also affect the nervous system. For instance, our previous study identified that the 53.7 THz (5.6 µm) wave could modulate the neuronal excitability through the voltage‐gated potassium channel, evidenced by electrophysiological recordings on native neurons and molecular dynamics simulations.^[^ ^14e^ ^]^ Moreover, we reported that the attenuation coefficient of the 53.7 THz wave in artificial cerebrospinal fluid (ACSF) is 0.0012 µm^−1^,^[^ ^14e^ ^]^ and that the effective penetration depth capable of inducing c‐Fos expression in neurons within the mouse brain is ≈400 µm.^[^ ^15d^ ^]^ In comparison, the attenuation coefficient of the 42.5 THz (0.0017 µm^−1^) wave in ACSF is similar to that of the 53.7 THz wave.^[^ ^14e^ ^]^ Therefore, we anticipate that the effective penetration depth of the 42.5 THz wave in brain tissue should be ≈300 µm. Experiments on mouse brains are necessary to explore the spatial extent of 42.5 THz stimulation, which is essential for clinical applications and future studies. While previous studies on terahertz modulation of ion channels and neurons have been substantial, most lack molecular validation, particularly in recombinant systems.^[^ ^16^ ^]^ In this study, we provided more convincing evidences from recombinant systems in HEK293 cells to minimize the confounding interplay of other membranal components and ascertain the precise regulatory roles of the 42.5 THz waves on targeted Ca_V_ channel. Complementing this, molecular dynamics simulations, Ca^2+^ imaging, and the strategic pharmacological isolation of neuronal Ca_V_1 currents collaboratively strengthen our conclusions. Our current experimental framework not only fortifies the credibility of our present conclusions but also paves the way for deeper insights into THz‐mediated biological effects in future studies.

## 4 Experimental Section

### Molecular Dynamics Simulation

The simulation system consists of a trimmed human voltage‐gated calcium channel (Ca_V_1.2, backbone from Alphafold Database, ID: AF‐Q13936, and improved with the PDB ID: 8FD7)^[^ ^17^ ^]^ embedded in bilayer lipid membranes (with the normal along the z direction), which are immersed in the CaCl_2_ solution with an ionic concentration of 0.15 mol L^−1^. To reduce the computing efforts, only the residues in the pore domain are considered in the simulations. The simulated pore protein is composed of four identical chains. Before the formal simulation, the system experiences energy minimization (EM), 375 ps temperature equilibration, and then 11.5 ns pressure equilibration to fully solvate mobile water and lipids around the protein. The free energy profile was calculated with WHAM method implemented in GROMACS based on the umbrella sampling. The detailed simulation settings can be found in Note S1 and Table S1 (Supporting Information).

### Plasmids and Viruses

Reconstitution of Ca_V_1.2 is based on the plasmids of α_1C_ (rabbit, X15539), β_2a_ (rat, M80545) and α_2_δ (rat, NM012919.2) in pcDNA3 vector.^[^ ^34^ ^]^ The sequence of CaMPARI2 is extracted from the addgene (Plasmid #101060).^[^ ^20^ ^]^ Following the practice of GCaMP‐X,^[^ ^35^ ^]^ the identical protective sequence CBM (Ca^2+^/Calmodulin binding motif) was added to the N‐terminus of CaMPARI2. The CBM sequence does not alter the Ca^2+^‐sensing properties but effectively prevents the probe from interacting with neuronal Ca_V_1 channels and ensures undistorted Ca^2+^/CREB signaling. The optimized CaMPARI2 was carried by an AAV viral vector with a neuron‐specific *Syn* promoter. The virus was produced by a local bio‐company (Hanbio Co., Ltd, Shanghai, China).

### Transfection of cDNA Constructs

The HEK293 cell line (ATCC) was free of mycoplasma contamination, checked by PCR with primers 5′‐GGCGAATGGGTGAGTAACACG‐3′ and 5′‐CGGATAACGCTTGCGACCTATG‐3′. The recombinant Ca_V_1.2 channels were transiently transfected according to an established calcium phosphate protocol.^[^ ^35^ ^]^ 5 µg of cDNA encoding α_1C_, 4 µg of β_2a_ and 4 µg of α_2_δ were applied to HEK293 cells in 60 mm dishes. cDNA of simian virus 40 T antigen (1 µg) was co‐transfected into HEK293 cells to enhance the expression of Ca_V_1.2 channels. Cells were washed with PBS 6 h after transfection and maintained in the cultured medium of DMEM (dulbecco's modified eagle medium) supplemented with FBS (Foetal Bovine Serum) and PS (penicillin‐streptomycin). Then cells were incubated for 2 days in 5% CO_2_ incubator at 37 °C before whole‐cell recordings.

### Whole‐Cell Electrophysiology

Whole‐cell patch‐clamp recordings of HEK293 cells transiently expressing recombinant Ca_V_1.2 were performed at room temperature (25 °C) using an Axopatch 700B amplifier (Molecular Devices) or a HEKA EPC10 Patch Clamp Amplifier (HEKA). Electrodes were pulled with borosilicate glass capillaries by a programmable puller (P‐1000, Sutter Instrument) and heat‐polished by a microforge (MF‐830, Narishige), resulting in 2–5 MΩ resistances before 70% of compensation. The internal/pipette solution contained (in mM): CsMeSO_3_, 135; CsCl, 5; MgCl_2_, 1; MgATP, 4; HEPES, 5; and EGTA, 5; with ≈290 mOsm adjusted with glucose and pH 7.3 adjusted with CsOH. The extracellular solution contained (in mM): TEA‐MeSO_3_, 135; HEPES, 10; CaCl_2_, 10; with ≈300 mOsm, adjusted with glucose and pH 7.3 adjusted with TEA‐OH, according to the previous protocols.^[^ ^35^ ^]^ Whole‐cell currents were evoked by repeated step depolarizations from a holding potential of −70 to +10 mV, or by a family of step depolarizations (−70 to +50 mV from a holding potential of −70 mV and step increase of 10 mV). All current traces were recorded at 2 kHz low‐pass filtering in response to voltage steps with a minimum interval of 30 s. Leak subtraction was used throughout. Ca^2+^ currents were normalized over different cells by cell capacitance (Cm, in pF). To quantify the CDI of calcium currents, the current amplitudes at peak, 50 or 300 ms were measured and marked as *I*_peak_, *I*_50_, and *I*_300_, respectively. The *r*_50_ = *I*_50_/*I*_peak_, and *r*_300_ = *I*_300_/*I*_peak_. To depict the normalized *I‐*‐*V* curves, all peak currents in the same family of step depolarizations were normalized to the maximum peak currents. To depict the voltage dependence of activation, all normalized data points in the same family of step depolarizations were fitted according to the following modified Boltzmann distribution: *I* = (*G*_max_∙(*V*‐*V*_rev_))/(1+exp((*V*_m_‐*V*)/*k*_s_), where *V* is the membrane potential, *I* is the normalized peak current, *G*_max_ is the maximum membrane conductance, *V*_rev_ is the extrapolated reversal potential, *V*_m_ is the voltage at half‐maximal activation, and *k*_s_ is the slope factor. Then summarized curves of voltage dependence of activation were fitted by a single Boltzmann distribution as below: *I*/*I*_max_ = A2+(A1‐A2)/(1+exp((*V*‐*V*_m_)/*k*_s_), where A1 and A2 being the normalized currents.

To fit the temporal profile of the peak Ca^2+^ currents, data points are separated into an on‐rate phase (colored as pink) and an off‐rate phase (colored as cyan). Both phases were fitted using the single Boltzmann distribution. The values of EC_50_ and IC_50_ are defined as the τ_on_ and τ_off_, respectively.

### THz Source

A pulsed quantum cascade laser (Daylight solutions, MIRcat‐QT Broadly‐Tunable Mid‐IR Laser) was used for THz stimulation. The laser emits radiation between 5 and 11 µm (range of pulse width: 100 to 500 ns; pulse repletion rate: 10 to 100 kHz). The laser contains four modules for continuous wavelength tuning but it was set to work constantly at 42.5 THz for stimulation. The laser output was coupled to an infrared fiber (Fiber Cable PIR240/300‐100‐FC/PC‐SP30, core 240 ± 15µm, NA = 0.30 ± 0.03) with a core diameter of 240 µm and a cladding diameter of 300 µm. For whole‐cell electrophysiology, the average power at the fiber tip (measured in the air) was at a stable level of 3 ± 0.3 mW with the following configurations: pulse duration 500 ns, repetition rate 100 kHz.

### Local Temperature Measurement

Before measurement, room‐temperature artificial cerebrospinal fluid was circulated throughout the entire system. Utilizing a 3D translation platform, both the fiber optic and temperature probe (TC‐334C Dual Channel Temperature Controller, Warner Instruments) were submerged in water. Under a microscope, the distance between the fiber optic port and the temperature probe was then adjusted. Once set at the desired distance, photographs were taken using the imaging software (IR capture). Temperature changes were recorded using the Clampex script. The recording protocol was as follows: initially record for 3 min without the laser, then activate the laser and record for an additional 10 min, and finally deactivate the laser, continuing the recording for roughly 5 min until the initial temperature is reached.

### Cultured Neuron Preparation

Cortical or hippocampal neurons were dissected from postnatal day 0 (P0, either sex) newborn ICR mice or Sprague‐Dawley rats. Isolated cortex tissues or hippocampus tissues were digested with 0.25% trypsin for 15–25 min at 37 °C, followed by terminating the enzymatic reaction by DMEM supplemented with 10% FBS. The suspension of cells was sieved through a filter and then centrifuged at 1000 rpm for 5 min. The cell pellet was resuspended in DMEM supplemented with 10% FBS and then plated on poly‐D‐lysine‐coated 35‐mm No. 1.5 confocal µ‐dishes (ibidi) to ensure the high transparency of 42.5 THz laser. After 4 h, neurons were maintained with Neurobasal medium supplemented with 2% B27, and 1% glutaMAX‐I (growth medium). Temperature of 37 °C with 5% CO_2_ was controlled in the incubator. The AAV‐*Syn*‐CaMPARI2 virus was added into the growth medium on P0. Animals were obtained from the Beijing Vital River Laboratory Animal Technology. All animal‐related procedures received approval from the ethical committees at Beihang University.

### CaMPARI2 In Vitro Photoconversion

The maximum intensity of 405 nm laser in the confocal microscopy (Andor Dragonfly 200 spinning disk confocal microscopy) was applied to cultured neurons for 1 min to trigger the green‐to‐red photoconversion of CaMPARI2 in the cultured cortical neurons from newborn ICR mice. The 488 and 561 nm waves were applied to excite the green fluorescence and red fluorescence to quantify the *F*_red_/*F*_green_. All data points were normalized to the control group (5 K or basal), in order to compare the total calcium influxes. Two or more independent culture preparations and n≥10 neurons for each experiment.

### Immunocytochemistry of Cultured Neurons

Neurons were maintained for 1.5 weeks before immunocytochemistry. The 5 K (5 mM [K^+^]_O_) solution consisted of 130 mm NaCl, 5 mm KCl, 1 mm MgCl_2_, 2 mm CaCl_2_, 15 mm HEPES, 10 mm glucose, pH 7.4 adjusted with NaOH. And the 40 K solution consisted of 95 mm NaCl, 40 mm KCl, 1 mm MgCl_2_, 2 mm CaCl_2_, 15 mm HEPES, 10 mm glucose, and pH 7.4 adjusted with NaOH. The 5 K solution represents the standard extracellular potassium concentration of 5 mm, which is typically found under physiological conditions. In contrast, the 40 K solution is a high‐potassium extracellular solution with an elevated potassium concentration of 40 mm. This increase in extracellular potassium reduces the potassium gradient across the neuronal membrane, leading to a partial depolarization of the resting membrane potential to ≈−19 mV.^[^ ^19^ ^]^ As a result, neurons become more excitable and are sensitized to spontaneous firing. The 40 K stimulus is a conventional approach to trigger Ca^2^⁺ influx predominantly through Ca_V_1 channels. For immunostaining of neurons under high potassium stimulation, 5 and 40 K solutions were supplemented with 0.5–1 µm TTX to block the sodium channels, 10 µm NBQX to block the AMPA receptors, 10 µm APV to block the NMDA receptors, 10 µm isradipine to block the Ca_V_1 channels (optional), before usage.^[^ ^19^ ^]^ 0.5–1 µm TTX was applied to neurons for 4–6 h to suppress the action potentials. Then neurons were maintained with 5 K solution for 5 min. 40 K, THz stimulation, and isradipine treatment were separately or jointly applied to neurons for 5 min. And neurons were fixed for pCREB and CREB immunostaining after 30 min. For immunostaining of neurons under physiological condition, neurons were maintained in 5 K solutions supplemented with 2% B27, and 1% glutaMAX‐I. Then 5 min THz stimulation, or 5 min THz stimulation along with 10 µm isradipine (pretreated with neurons for 5 min before THz stimulation), were applied to neurons. Neurons were fixed for pCREB, CREB, and c‐Fos immunostaining after 30 min. All neurons on confocal dishes were rapidly rinsed with 4 °C PBS and then fixed with 4 °C 4% paraformaldehyde in PBS for 15–20 min. Fixed neurons were washed by PBS for 3 times, permeabilized with 0.3% Triton X‐100 for 5 min, blocked by 10% goat serum in PBS for 1 h, and incubated with primary antibodies overnight at 4 °C. Information for primary antibodies: pCREB (Rabbit mAb #9198, Cell Signaling Technology, Species Cross‐Reactivity: Human, Mouse, Rat, Dilutions: 1:500 in PBS), CREB (Rabbit mAb #9197, Cell Signaling Technology, Species Cross‐Reactivity: Human, Mouse, Rat, Monkey, D. melanogaster, Dilutions: 1:500 in PBS), c‐Fos (Rabbit mAb [EPR21930‐238] #ab222699, Abcam, Species Cross‐Reactivity: Mouse, Human, Dilutions: 1:1000 in PBS). The next day, neurons were washed with PBS for three times, incubated with the secondary antibodies (Goat anti‐Rabbit Alexa Fluor 647, #4414, Cell Signaling Technology, Dilutions: 1:800 in PBS) for 2 h, and treated with Hoechst 33342 (Invitrogen) for nuclear counterstain for 5 min. Mounted neurons on confocal dishes were imaged with a confocal microscope (Andor Dragonfly 200 spinning disk confocal microscopy or Leica SP8 confocal microscopy). Nuclear immunofluorescence was analyzed by FIJI. The immunostaining for pCREB and CREB was conducted on cultured neurons from newborn ICR mice, while the c‐Fos immunostaining was carried out on neurons from newborn SD rats. Two or more independent culture preparations and n≥12 neurons for each experiment.

### Ca^2+^ Imaging

The cultured cortical neurons virally expressing CaMPARI2 were subjected to Ca^2+^ imaging on confocal microscopy (Andor Dragonfly 200 spinning disk confocal microscopy). Sampling rate: 5 Hz. Excitation: 488 nm laser. Emission: 510 nm. CaMPARI2 could act as a green genetically‐encoded Ca^2+^ indicator that shows decreased fluorescence with increasing Ca^2+^ levels if without the PC light photoconversion.^[^ ^36^ ^]^ 30 s THz stimulation was applied to neurons to evoke the calcium responses. At least four trials were recorded. Then neurons were treated with 10 µm isradipine for 5 min and recorded for at least four trials. Finally, neurons were washed with cultured medium for three times and recorded for one more trial. More than two independent experiments were performed. Fluorescence intensity (*F*) was background‐subtracted. *F*_0_ was the baseline fluorescence averaged from at least 1 s. ‐*F*/*F*_0_ serves as the index to represent the calcium dynamics. Two or more independent culture preparations for the experiments.

### Immunocytochemistry of Brain Slices

C57BL/6J male mice (2–3 months old, 6 mice in total) were placed in an anesthesia box for a 30‐min adaptation period. Subsequently, the gas anesthesia was activated to gradually induce sleep. An intraperitoneal injection of 10% Chloral hydrate (0.08 mL/10 g dosage) was administered. The fiber tip was strategically positioned over the right brain surface through an open craniotomy. Three mice received direct stimulation to the right brain using a 42.5 THz wave. Meanwhile, another three mice were pre‐treated with preheated artificial cerebrospinal fluid (129 mM NaCl, 3 mM KCl, 1.3 mM MgSO_4_, 1.2 mM KH_2_PO_4_, 3 mM HEPES, 10 mM Glucose, 20 mM NaHCO_3_, 2.4 mM CaCl_2_) containing 10 µm isradipine for 5 min prior to the THz stimulation of their right brain. Post‐THz stimulation, the mice were maintained under anesthesia for an hour to facilitate c‐Fos expression. Mice were then perfused transcardially using 4% paraformaldehyde (PFA). The brain was extracted, fixed with 4% PFA, and stored at 4 °C overnight. Coronal sections, 40 µm in thickness, were produced. c‐Fos immunostaining was done using the primary antibody Anti‐c‐Fos (1:500 dilution) and followed by the respective secondary antibody (1:500 dilution). DAPI staining (1:50 dilution) was employed for cell nuclei identification. Cells that displayed both c‐Fos and DAPI were categorized as c‐Fos positive, while those showing only DAPI were marked as c‐Fos negative. Three mice for each experiment.

### THz Stimulation of Mouse Brain

Three two‐month‐old C57BL/6J male mice were anesthetized with 10% Chloral hydrate (dosage: 0.1 mL/10 g), and then drilled holes in the primary motor cortex. After two days of culture, the 3 mice were lightly anesthetized with isoflurane, fixed to the brain stereotaxic apparatus, and when they woke up, they were treated with preheated artificial cerebrospinal fluid, and then the artificial cerebrospinal fluid is wiped clean with a cotton swab. Finally, the mouse's responses to THz stimulation were recorded using a mobile phone camera.

### Structural Analysis and RMSD Quantification

The amino acid sequences of the ten Ca_V_ subtypes (Ca_V_1.1‐Ca_V_1.4, Ca_V_2.1‐Ca_V_2.3, Ca_V_3.1‐Ca_V_3.3) were from the UniProt IDs: P07293, Q13936, Q01668, O60840, O00555, Q00975, Q15878, O43497, O95180 and Q9P0×4. The sequence of Ca_V_Ab was obtained from the PDB ID: 4MVQ. UniProt IDs of the Na_V_1.2, Na_V_1.4, and Na_V_1.5 channels: Q99250, P35499 and Q14524. UniProt IDs of the K_V_1.2, KCNQ1, and KcsA channels: P63141, Q9Z0N7, and P0A334. UniProt IDs of the TRPV1 and TRPC5 channels: Q8NER1 and Q9UL62. The structural information for Ca_V_1.1, Ca_V_1.2, Ca_V_1.3, Ca_V_1.4, Ca_V_2.2, Ca_V_2.3, Ca_V_Ab, K_V_1.2, TRPC5 and Na_V_1.4 was derived from PDB/AlphaFold IDs: 7JPV, AF‐Q13936, 7UHG, AF‐O60840, 7MIY, 6KZP, 4MVQ, 2A79, 7WDB and 6AGF, respectively. The RMSD values were calculated using PyMOL in all‐atom model. All images of structures were prepared using the PyMOL 2.4.1, ensuring accuracy and consistency in the visualization and quantification of the structural variances among the structures.

### Apoptosis Detection Assay

The Annexin V‐EGFP/PI Apoptosis Detection Kit (Cat #: KTA0005, Abbkine) was utilized. To establish a positive control, cultured neurons were treated with apoptosis inducers A and B from the kit. The neurons on confocal dishes were then exposed to 42.5 THz radiation for 5 min to evaluate both immediate and longer‐term effects of THz exposure, assessed after 2 h and 1 day, respectively. Following treatments, neurons were washed twice with PBS and incubated with Annexin V‐EGFP and PI for 15 min. Subsequent to nuclear staining by Hoechst and another washing step, the neurons were prepared for confocal microscopy analysis, with each experiment replicated at least twice and n≥12 neurons analyzed per experiment.

### RNA‐seq and Data Analysis

Tissue samples were individually collected from the brains of 3 irradiated mice and their corresponding controls. RNA extraction was performed using TRIzol reagent (Invitrogen, USA), and the RNA concentration and integrity were assessed using a NanoDrop 2000 microspectrophotometer (Thermo Fisher Scientific) and an Agilent 2100 Bioanalyzer (LabChip GX). A total of 2 µg of RNA, with an OD260/280 ratio within 1.7 to 2.5 and an RIN value of ≥ 7, was purified using mRNA Capture Beads. DNA libraries were then prepared and sequenced on an Illumina NovaSeq 6000 system (Illumina, San Diego) following the manufacturer's guidelines. Sequencing quality was evaluated using the Qsep‐400 system to generate FastQ data. Raw sequences were processed into clean reads, and the Hisat2 software tool was utilized for mapping to the reference genome. Gene expression levels were quantified in terms of fragments per kilobase of transcript per million mapped fragments (FPKM). Differentially expressed genes (DEGs) were identified with a threshold set at FDR < 0.05 and |log2(fold change)| ≥ 1 for significant differential expression. Gene Set Enrichment Analysis (GSEA) of the C5 (Gene Ontology gene sets) was conducted to analyze GO gene sets, employing the clusterProfiler software package in R.

### Statistical Analysis

Data were analyzed in Matlab, GraphPad Prism, Excel, OriginPro, Hisat2, and ClusterProfiler software. All data were shown as mean ± Standard error of the mean (S.E.M.). Student's *t*‐test, One‐way ANOVA, and Kruskal‐Wallis test (criteria of significance: ^*^ *p* < 0.05; ^**^ *p* < 0.01, ^***^ *p* < 0.001) were calculated when applicable, and *n.s*. denotes “not significant”.

## Conflict of Interest

The authors declare no conflict of interest.

## Author Contributions

C.C. and Y.Y. conceived the project. Y.Y. designed the experiments. C.F., Y.G., C.C., and Y.Y. supervised the work. Y.S., Y.F., Y.Z., J.C., and Y.Y. performed the experiments. Y.S. performed the simulation with the help of Y.L. and S.W. Y.S. and Y.Y. analyzed the data. Y.Y. wrote the manuscript. All authors participated in the revision of the manuscript. J.G. and other members in the lab of X.L. provided the crucial (both experimental and conceptual) paradigm established for bidirectional modulation of the excitation‐neuritogenesis coupling with Ca_V_1‐binding proteins and peptides (unpublished).

## Supporting information



Supporting Information

## Data Availability

The data that support the findings of this study are available from the corresponding author upon reasonable request.
