# Supplementary material for: A Non‐Invasive and DNA‐free Approach to Upregulate Mammalian Voltage‐Gated Calcium Channels and Neuronal Calcium Signaling via Terahertz Stimulation
Source: Adv Sci (Weinh). 2024 Oct 22;11(47):2405436. doi: 10.1002/advs.202405436 (PMC11653603; doi:10.1002/advs.202405436)
Supplement: Supplementary file 1 — Supporting Information [file ADVS-11-2405436-s001.docx]

Supporting Information

**A Non-Invasive and DNA-free Approach to Upregulate Mammalian Voltage-Gated Calcium Channels and Neuronal Calcium Signaling via Terahertz Stimulation**

Yuankun Sun, Jinli Geng, Yu Fan, Yangmei Li, Yuan Zhong, Jing Cai, Xiaodong Liu*, Shaomeng Wang, Yubin Gong,* Chao Chang,* Yaxiong Yang,* Chunhai Fan

Table of contents

Supplementary Text 1 | The detailed simulation settings and calculations.

Table S1. The parameter settings for different objects being restrained in different simulation phases.

Figure S1. Additional information for model building of Ca_V_1.2.

Figure S2. Additional information for calculation of free energy.

Figure S3. Additional information for the SF regions across Ca_V_ family and Ca_V_Ab.

Figure S4. Additional information for the THz source and THz-mediated temperature elevation.

Figure S5. Additional information for the immunofluorescent signals of cultured cortical neurons treated with blocker cocktail.

Figure S6. Additional information for the immunofluorescent signals of cultured cortical neurons in basal condition.

Figure S7. Additional information for the immunofluorescent signals in cultured hippocampal neurons.

Figure S8. THz induced behavioral responses in the awake mice.

Figure S9. RNA-seq supplementary data.

Supplementary Text 1 | The detailed simulation settings and calculations

The detailed simulation settings.

In the simulations, the LINCS constraint algorithm was used to convert all covalent bonds with H-atoms to rigid holonomic constraints, so the integration time step was set to 2 fs. The non-bonded interactions such as Van der Waals (vdw) and electrostatic interactions were calculated on the basis of a neighbor list which is generated by the Verlet algorithm and was updated every 20 fs. The short-range electrostatic interactions were computed within a cutoff distance of 12 Å, beyond that the long-range method Particle Mesh Ewald (PME) summation was used, and the vdw forces were calculated within a cutoff of 12 Å.

To ensure the stability of the initial structure, an energy minimization was first performed. Then the weak coupling method (berendsen) was implied to scale the temperature to a bath of 315 K, which was named as the NVT equilibration. The semi-isotropic Berendsen barostat algorithm was used to maintain the pressure constantly at 1 bar (NPT equilibration). After the simulation system was equilibrated sufficiently, the more accurate Nose-Hoover thermostat and semi-isotropic Parrinello-Rahman barostat algorithms were adopted.

During equilibration, to ensure that the atoms of proteins and lipids were harmoniously confined to fixed reference positions, positional and dihedral constraints were applied. This was to avoid drastic rearrangements of critical parts. After that, a 10 ns non-restrained NPT process had been performed to obtain reasonable conformations of protein and lipids. The parameter settings were given in Table S1.

**Calculation of the oscillation spectra of the C-O bond length**

A molecular dynamics simulation of 700ps was conducted. In the simulation, the integration time step was set as 1fs, and the non-bonded interactions was updated every 4 fs. Two groups were defined in the index file, including the C atom and O atom from -COO^-^. Then the distance instruction of GROMACS was used to extract the -C=O band length from the -COO^-^ group. Finally, the bond length data was imported into Matlab to calculate its vibration spectrum. In the Matlab script, the sampling interval was 4 fs, which means the sampling frequency was 1/4 fs.

**Calculation of the absorption spectra of the selectivity filter**

The absorption peak was calculated by Gaussian. The selective filter was extracted from the balanced gro file. Water was added as a solvent in the calculation, and the base group used was def2-TZVP. In the simulation, only the residues in the selective filter were considered in the calculations to reduce the computing efforts. Finally, the calculated frequency was corrected by the correction coefficient.

Table S1. The parameter settings for different objects being restrained in different simulation phases.

| Phase | Simulation time | Position restraints | | | |
| --- | --- | --- | --- | --- | --- |
|  |  | Four protein chains | | Lipids (POPC) | |
|  |  | backbone | Side chains | P atom | dihedral |
|  |  | (kJ/mol/nm^2^) | (kJ/mol/nm^2^) | (kJ/mol/nm^2^) | (kJ/mol/nm^2^) |
| EM | / | 4000 | 2000 | 1000 | 1000 |
| NVT1 | 125 ps | 4000 | 2000 | 1000 | 1000 |
| NVT2 | 125 ps | 2000 | 1000 | 1000 | 400 |
| NVT3 | 125 ps | 1000 | 500 | 400 | 200 |
| NPT1 | 500 ps | 500 | 200 | 200 | 200 |
| NPT2 | 500 ps | 200 | 50 | 40 | 100 |
| NPT3 | 500 ps | 200 | 50 | 20 | 50 |
| NPT4 | 10 ns | 0 | 0 | 0 | 0 |
| Pulling | 5.0 ns | 200 | 50 | 1000 | 400 |
|  |  | except for all SF residues | |  |  |
| NPT at US | 1.0 ns | 200 | 50 | 1000 | 400 |
|  |  | except for all SF residues | |  |  |
| MD at US | 3.0 ns | 200 | 50 | 1000 | 400 |
|  |  | except for all SF residues | |  |  |

**
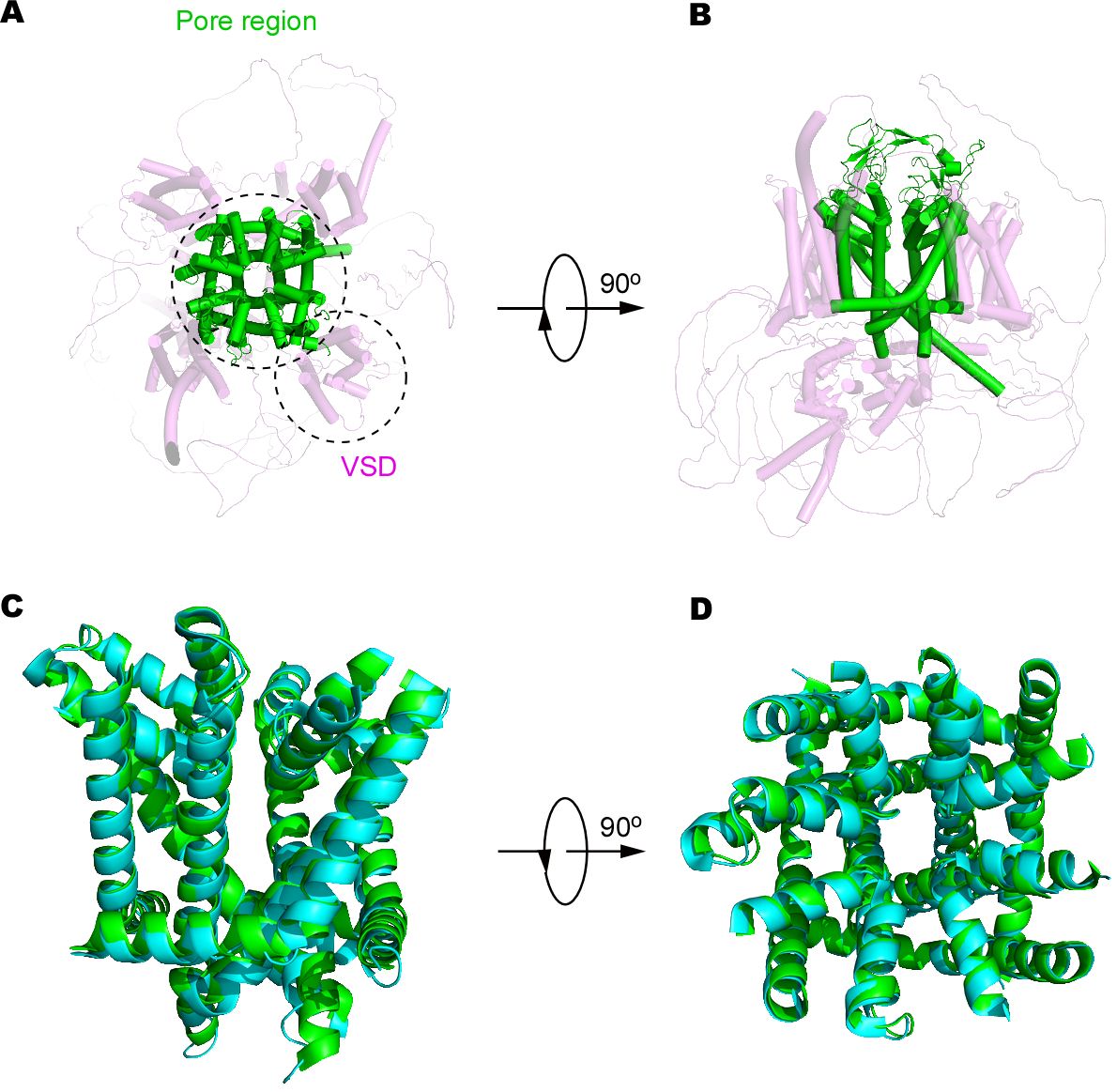
**

**Figure S1. Additional information for model building of Ca_V_1.2.**

**(A)** The top view of a human α_1C_ structure from Alphafold Database, ID: AF-Q13936. All helices are shown as cylinders. The pore region and voltage-sensing domains (VSD) are circled, and colored in green and pink, respectively.

**(B)** The side view of the human α_1C_ structure.

**(C)** The side view of a comparison of the human α_1C_ pore regions between the structure from Alphafold Database, ID: AF-Q13936 (green) and the structure from the PDB ID: 8FD7 (in cyan). The root-mean-square deviation (RMSD) of the two structures is 1 Å, measured by PyMOL.

**(D)** The top view of the comparison.

**
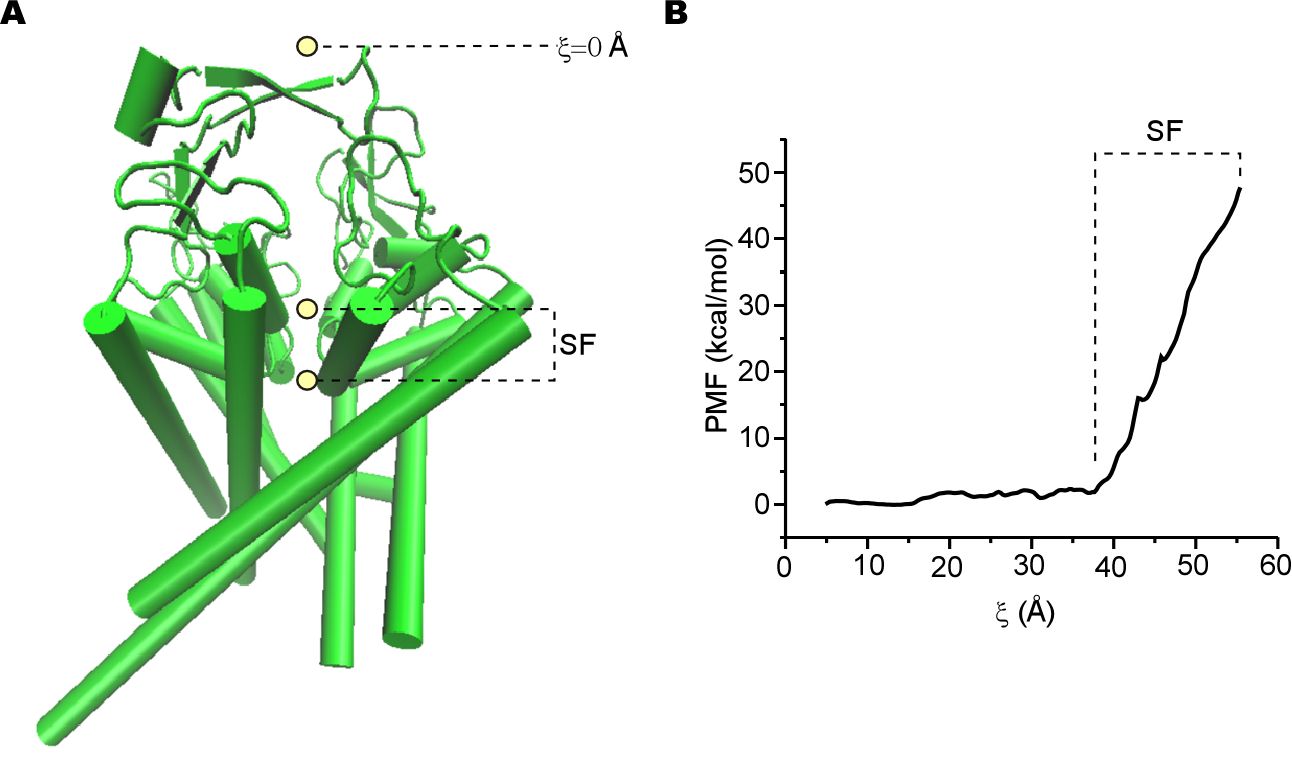
**

**Figure S2.** **Additional information for calculation of free energy.**

**(A)** The pore region of a modified human α_1C_. The calcium ions are colored in yellow. The starting position (ξ = 0 Å) and the selective filter (SF) are labelled.

**(B)** Free energy profile without the THz stimulation. The curve representing the SF is labeled.


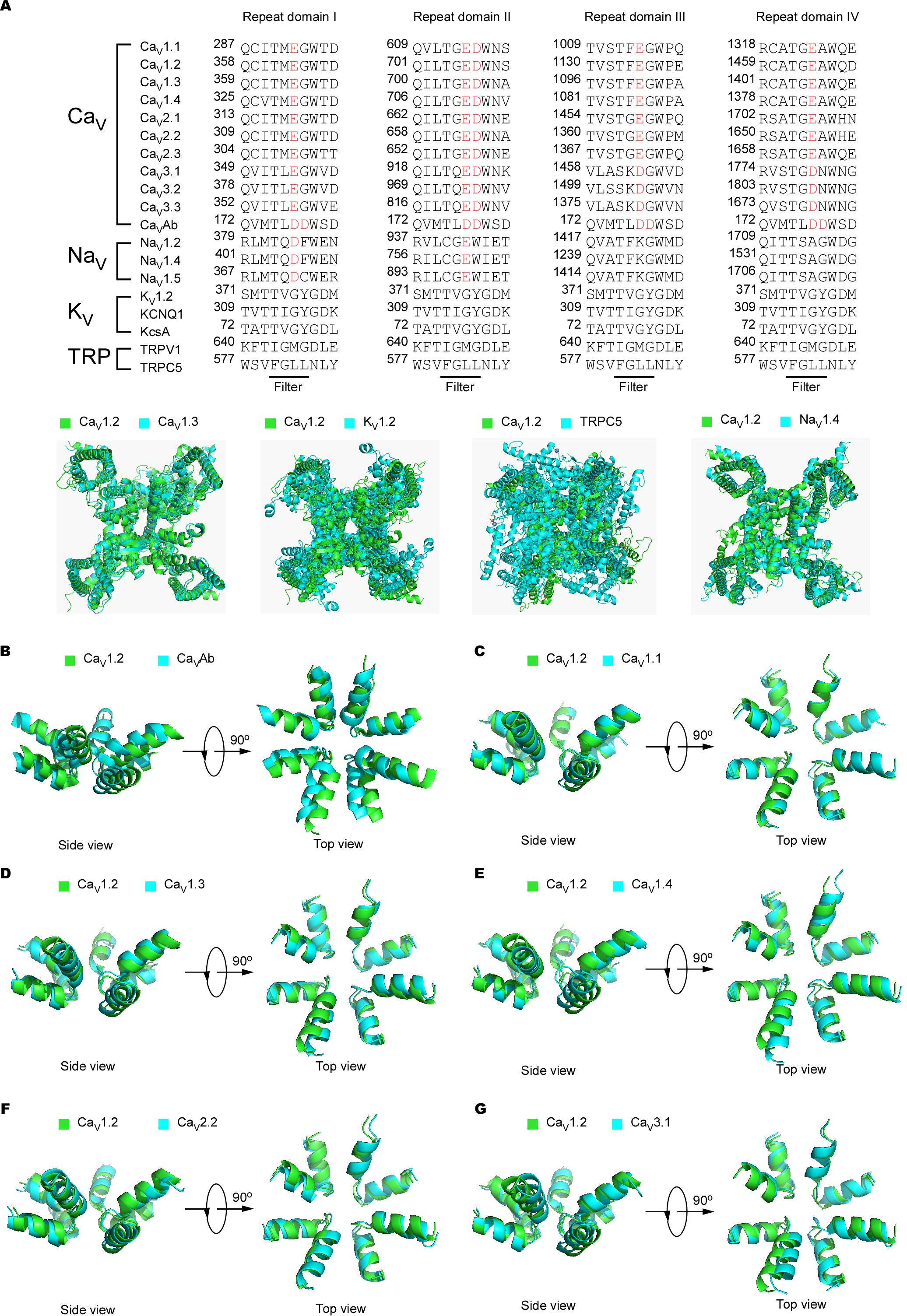


**Figure S3. Additional information for the SF regions across Ca_V_ family and Ca_V_Ab.**

**(A)** Multiple sequence alignment of the SF regions across the ten subtypes in Ca_V_ family and the Ca_V_Ab, the representative Na_V_ channels, the representative K_V_ channels, and the representative TRP channels. UniProt IDs of the ten Ca_V_ subtypes from Ca_V_1.1 to Ca_V_3.3: P07293, Q13936, Q01668, O60840, O00555, Q00975, Q15878, O43497, O95180 and Q9P0X4. The sequence of Ca_V_Ab is from the PDB ID: 4MVQ. UniProt IDs of the 3 representative Na_V_ channels: Q99250, P35499 and Q14524. UniProt IDs of the 3 representative K_V_ channels: P63141, Q9Z0N7 and P0A334. UniProt IDs of the 2 representative TRP channels: Q8NER1 and Q9UL62. The key amino acids in SF core regions are underlined. The negative charged amino acids in the SF core regions are colored in red. For comparisons between Ca_V_1.2 and other channels. The pore-forming subunit of Ca_V_1.2 is colored in green and other channels are colored in cyan. The PDB IDs of other channels: Ca_V_1.3: 7UHF, K_V_1.2: 2A79, TRPC5: 7WDB and Na_V_1.4: 6AGF.

**(B-G)** Structural comparisons of the SF regions between Ca_V_1.2 and other Ca_V_ channels. Ca_V_1.2 are colored in green and other Ca_V_ channels are colored in cyan. The side views and top views are shown. Ca_V_2.2 and Ca_V_3.1 serve as the representative channels for Ca_V_2 and Ca_V_3. The PDB/AlphaFold IDs of Ca_V_1.1, Ca_V_1.3, Ca_V_1.4, Ca_V_2.2, Ca_V_3.1 and Ca_V_Ab: 7JPV, 7UHG, AF-O60840, 7MIY, 6KZP and 4MVQ.


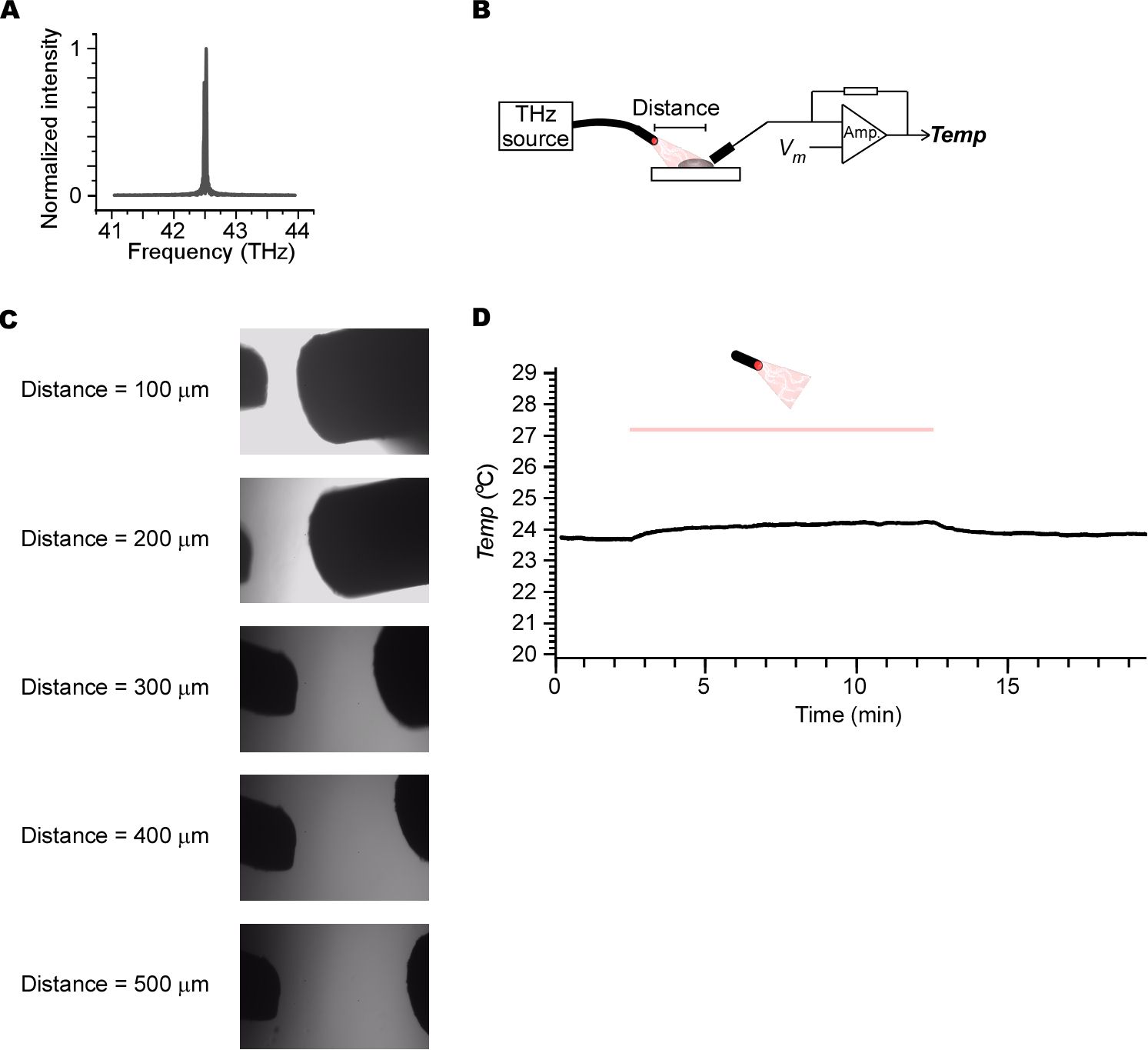


**Figure S4. Additional information for the THz source and THz-mediated temperature elevation.**

**(A)** The spectrum of 42.5 THz stimulation from the THz source. The intensities at varying frequencies were measured by Nicolet 8700 FTIR spectrometer (Thermo Scientific) and normalized to the maximum intensity.

**(B)** Configuration for temperature recording. A carbon fiber electrode was utilized to record the local temperature elevation.

**(C)** Images to show the configuration of temperature recording. The distances between the optical fiber port and the glass electrode vary from 100 μm to 500 μm.

**(D)** Representative trace of temperature recording. The distance between the optical fiber port and the glass electrode is 300 μm. 10 min THz irradiation is labeled.


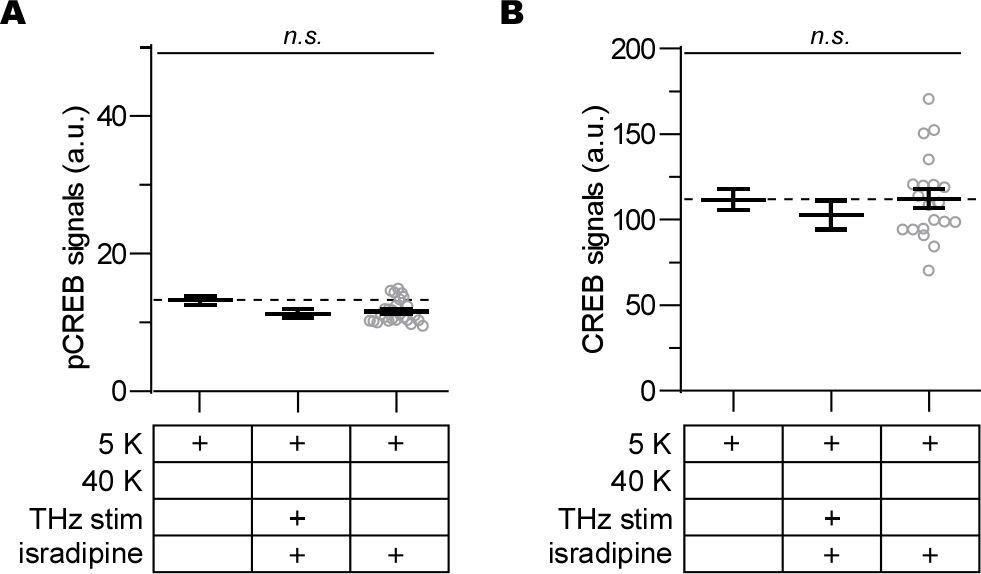


**Figure S5. Additional information for the immunofluorescent signals of cultured cortical neurons treated with blocker cocktail.**

**(A, B)** Statistical summary of pCREB (**A**) and CREB (**B**) immunofluorescent signals for the cultured cortical neurons treated with the blocker cocktail in **Figure 4A**. Data of neurons under 5 K or under 5 K along with THz stim and isradipine treatment in (**A**) and (**B**) were from the **Figure 4C** and **Figure 4D**, respectively. Data of neurons under 5 K and isradipine treatment were generated from the same batch of neurons as the first two groups (n=26 cells for **A**, and n=20 cells for **B**). Isradipine alone did not alter the pCREB and CREB level for neurons treated with blocker cocktail.

Values are presented as mean±SEM. One-way ANOVA followed by Tukey for post hoc tests was used (*n.s.*, not significant, *p*>0.05).


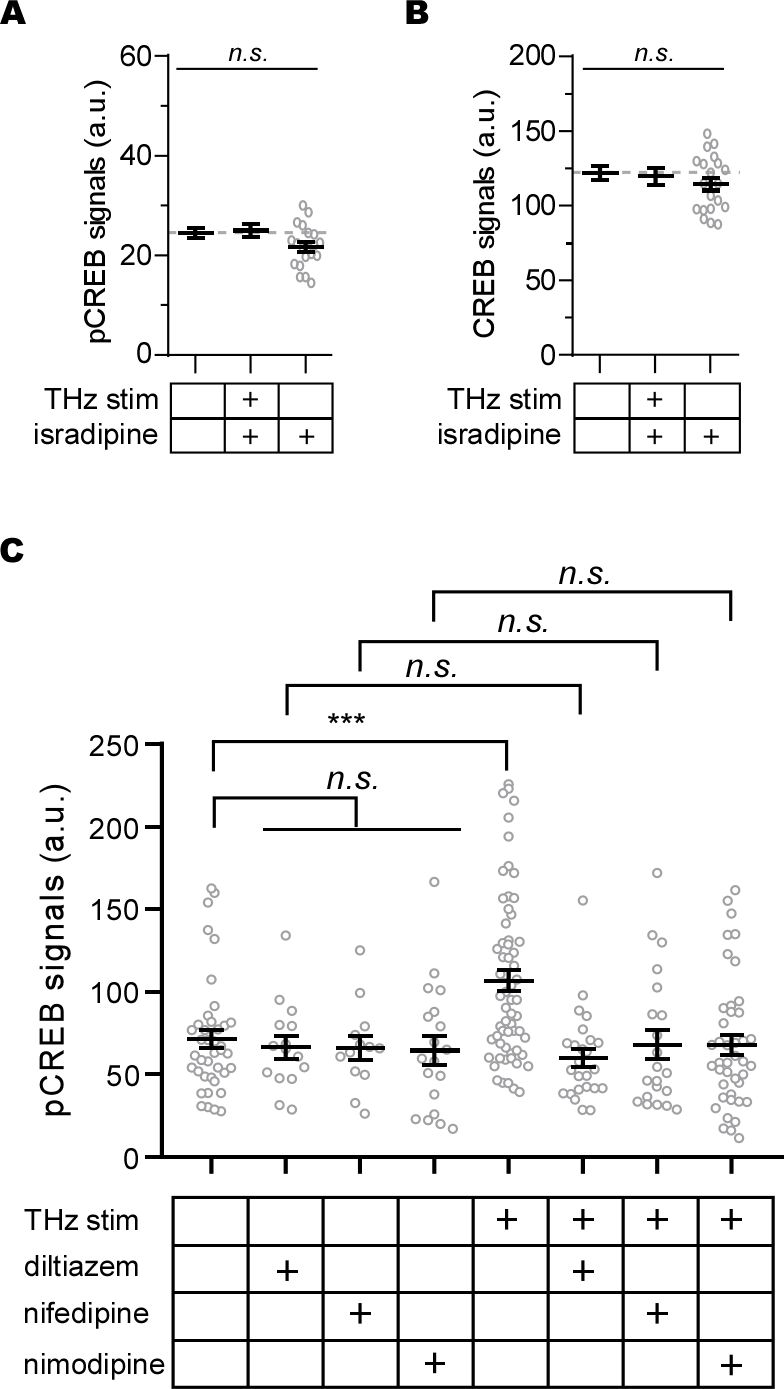


**Figure S6. Additional information for the immunofluorescent signals of cultured cortical neurons in basal condition.**

**(A, B)** Statistical summary of pCREB (**A**) and CREB (**B**) immunofluorescent signals for the cultured cortical neurons in basal condition. Data of neurons without THz stimulation or under THz stimulation along with isradipine treatment in (**A**) and (**B**) were from the **Figure 5D** left panel and **Figure 5D** right panel, respectively. Data of neurons under isradipine treatment alone were generated from the same batch of neurons as the first two groups (n=20 cells for **A**, and n=20 cells for **B**). Isradipine alone did not alter the pCREB and CREB level for neurons in basal condition.

**(C)** Statistical summary of pCREB immunofluorescent signals for the cultured cortical neurons in basal condition. These neurons were treated with three types of calcium channel blockers, both individually and in combination with 42.5 THz stimuli. (n=40, 15, 13, 19, 62, 25, 21 and 42 cells for 8 columns from left to right).

Values are presented as mean±SEM. One-way ANOVA followed by Tukey for post hoc tests was used (*n.s.*, not significant, *p*>0.05).


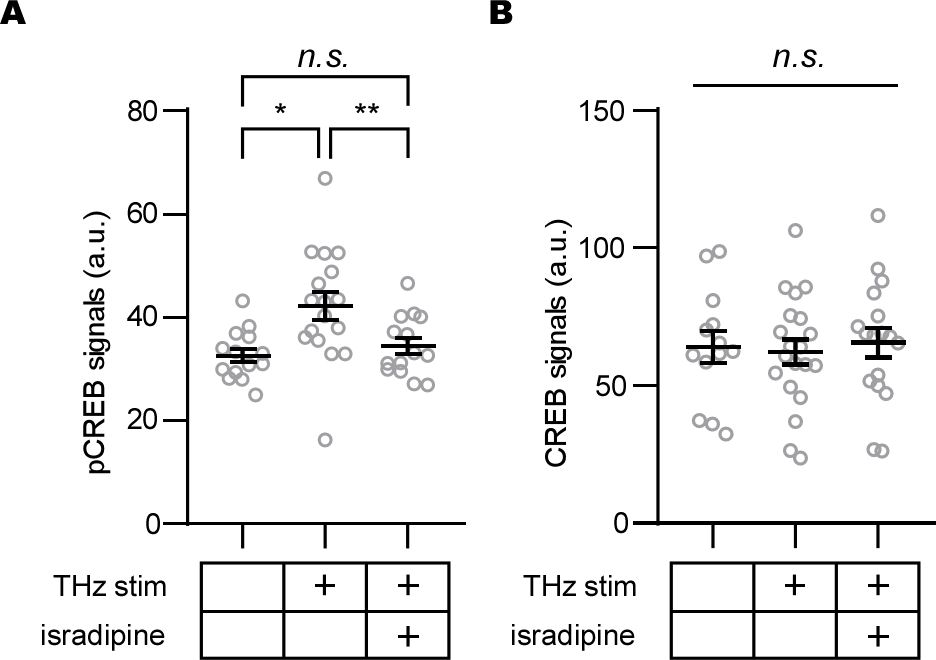


**Figure S7. Additional information for the immunofluorescent signals in cultured hippocampal neurons.**

**(A)** Statistical summary of pCREB signals for the cultured hippocampal neurons under 3 conditions: basal/physiological condition (n=14 cells), 42.5 THz stimuli (n=17 cells), and 42.5 THz stimuli under isradipine treatment (n=14 cells).

**(B)** Statistical summary of CREB immunofluorescent signals for the neurons under the identical 3 conditions (n=13, 20, and 17 cells).

Values are presented as mean±SEM. One-way ANOVA followed by Tukey for post hoc tests was used (**p*<0.05; ***p*<0.01; *n.s.*, not significant, *p*>0.05).

**
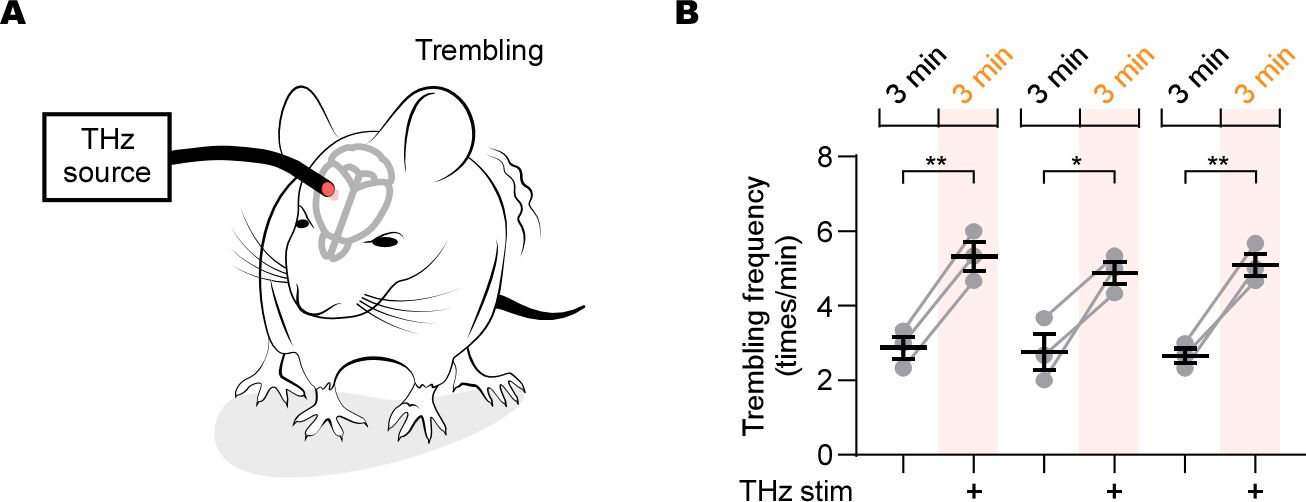
**

**Figure S8. THz induced behavioral responses in the awake mice.**

**(A)** Diagram showcasing mouse trembling induced by 42.5 THz stimulation of the awake mouse brain.

**(B)** Statistical summary of trembling frequency of awake mouse before and during THz stimulation (n=3 mice). Mouse were repeatably stimulated 3 times.

Values are presented as mean±SEM. Paired *t*-test (**B**) were used (**p*<0.05, ***p*<0.01).

**
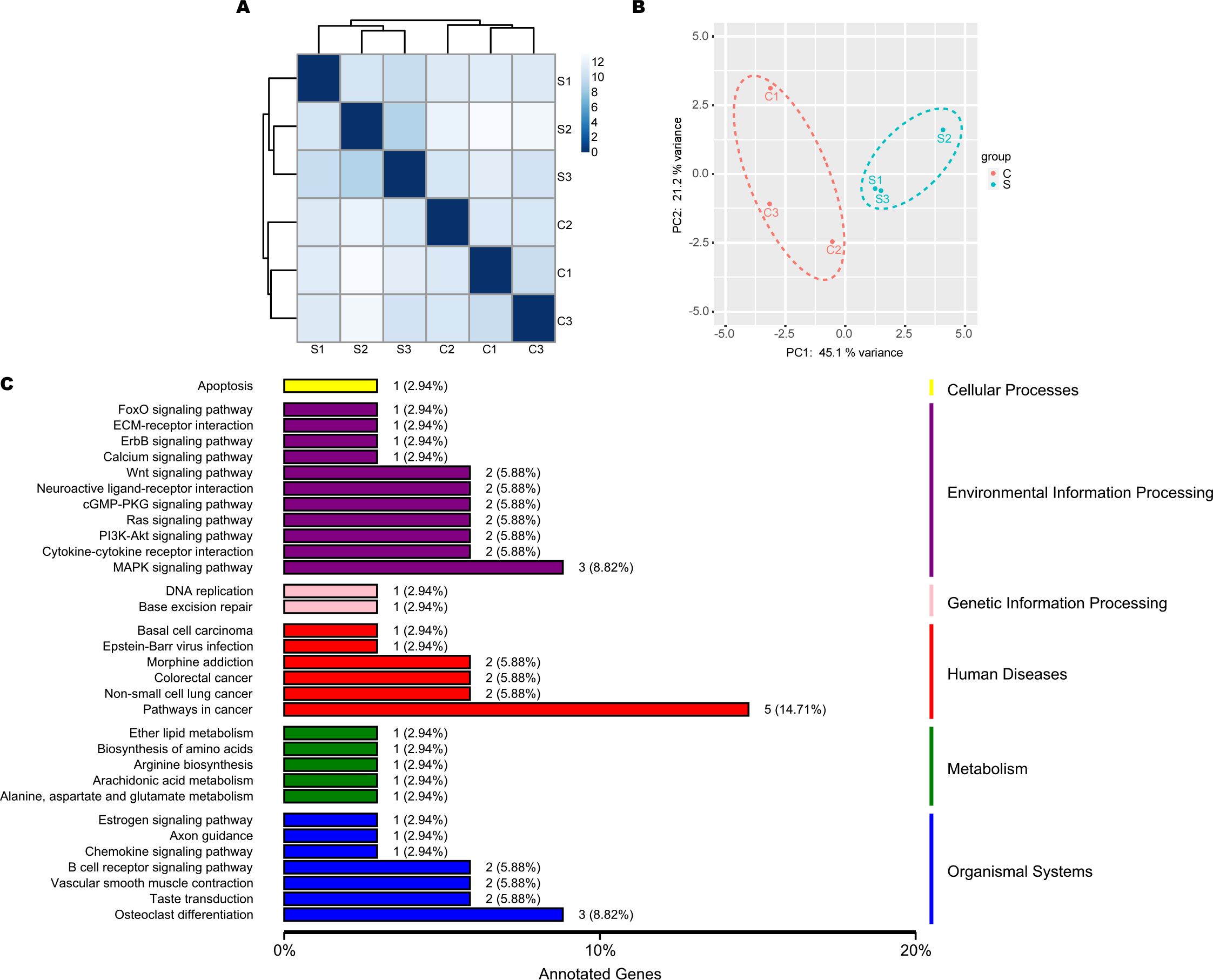
**

**Figure S9. RNA-seq supplementary data.**

**(A)** Heatmap of expression correlation between pairwise samples. C1, C2 and C3 represent the 3 control mice, and the S1, S2 and S3 represent the 3 mice under 42.5 THz stimuli.

**(B)** Principal component analysis of RNA-seq data from the 3 control mice (C1 to C3) and 3 mice under 42.5 THz stimuli (S1 to S3).

**(C)** The KEGG (Kyoto Encyclopedia of Genes and Genomes) classification analysis from the 3 control mice and the 3 stimulated mice.
